# Gene expression based inference of cancer drug sensitivity

**DOI:** 10.1038/s41467-022-33291-z

**Published:** 2022-09-27

**Authors:** Smriti Chawla, Anja Rockstroh, Melanie Lehman, Ellca Ratther, Atishay Jain, Anuneet Anand, Apoorva Gupta, Namrata Bhattacharya, Sarita Poonia, Priyadarshini Rai, Nirjhar Das, Angshul Majumdar, Jayadeva, Gaurav Ahuja, Brett G. Hollier, Colleen C. Nelson, Debarka Sengupta

**Affiliations:** 1 Department of Computational Biology, Indraprastha Institute of Information Technology-Delhi (IIIT-Delhi), Okhla, Phase III, New Delhi, 110020 India; 2 Australian Prostate Cancer Research Centre-Queensland, Faculty of Health, School of Biomedical Sciences, Centre for Genomics and Personalised Health, Queensland University of Technology, Translational Research Institute, Brisbane, QLD Australia; 3 Vancouver Prostate Centre, Department of Urologic Sciences, University of British Columbia, Vancouver, BC Canada; 4 Department of Computer Science and Engineering, Indraprastha Institute of Information Technology-Delhi (IIIT-Delhi), Okhla, Phase III, New Delhi, 110020 India; 5 Department of Biotechnology, Delhi Technological University, Shahbad Daulatpur, Main Bawana Road, Delhi, 110042 India; 6 Department of Electrical Engineering, Indian Institute of Technology Delhi, Hauz Khas, Delhi, 110016 India; 7 Centre for Artificial Intelligence, Indraprastha Institute of Information Technology-Delhi (IIIT-Delhi), Okhla, Phase III, New Delhi, 110020 India; 8 Department of Electronics & Communications Engineering, Indraprastha Institute of Information Technology-Delhi (IIIT-Delhi), Okhla, Phase III, New Delhi, 110020 India

**Keywords:** Cancer therapeutic resistance, Computational models, Machine learning, Cancer genomics, Cancer models

## Abstract

Inter and intra-tumoral heterogeneity are major stumbling blocks in the treatment of cancer and are responsible for imparting differential drug responses in cancer patients. Recently, the availability of high-throughput screening datasets has paved the way for machine learning based personalized therapy recommendations using the molecular profiles of cancer specimens. In this study, we introduce Precily, a predictive modeling approach to infer treatment response in cancers using gene expression data. In this context, we demonstrate the benefits of considering pathway activity estimates in tandem with drug descriptors as features. We apply Precily on single-cell and bulk RNA sequencing data associated with hundreds of cancer cell lines. We then assess the predictability of treatment outcomes using our in-house prostate cancer cell line and xenografts datasets exposed to differential treatment conditions. Further, we demonstrate the applicability of our approach on patient drug response data from The Cancer Genome Atlas and an independent clinical study describing the treatment journey of three melanoma patients. Our findings highlight the importance of chemo-transcriptomics approaches in cancer treatment selection.

## Abstract (web summary)

Predicting treatment response in cancer remains a highly complex task. Here, the authors develop Precily, a deep neural network framework to predict treatment response in cancer by considering gene expression, pathway activity estimates and drug features, and test this method in multiple datasets and preclinical models.

## Introduction

Cancer is a highly complex disease exhibiting varying degrees of genetic and phenotypic heterogeneity within individuals. Despite the apparent overall improvement in prognosis, responses to cancer treatment are often unpredictable. This is primarily attributable to the clonal diversity of cancer cells and associated phenotypically altered non-malignant cells in the tumor microenvironment. These pose a substantial hindrance to the optimal therapeutic management of the disease^1,2^. Contemporary therapeutic strategies use cancer drugs, with lower toxicity that specifically target aberrantly expressed or mutated proteins and, in general. *EGFR* expression and mutations, *KRAS* mutations, *BCR-ABL* fusions, and *HER2* overexpression are such examples of common therapeutic targets in cancer^3^. Unfortunately, not all cancers and anti-cancer drugs are known to be associated with strong targetable genetic biomarkers. As such, it is concluded that the simple relationship of drug targets or mutational status alone is incomprehensive for predicting the efficacy of specific targeted therapies^4,5^. Furthermore, administering a targeted therapy without considering drug resistance as a consequence may lessen patient survival. The drug resistance might be manifested through clonal expansion under treatment-induced selective pressure or from alternative signaling pathways that sustain tumor growth^6^. As such, early inference of drug response based on pretreatment molecular portraits of cancer has become a necessity^2,7^.

In recent years, the availability of large-scale pharmacogenomic databases has propelled predictive personalized oncology research^2^. Cancer Cell Line Encyclopedia (CCLE),^8^ Genomics of Drug Sensitivity in Cancer (GDSC)^9^, and Cancer Therapeutics Response Portal v2 (CTRPv2)^10^ are noteworthy among these. These high-throughput screening studies constitute an expansive knowledge base comprising high-throughput screening studies entailing more than 1000 cell lines and several hundreds of anticancer drugs^2^. Concurrently, The Cancer Genome Atlas (TCGA)^11^ serves as another rich database featuring gene expression profiles of mostly primary tumors spanning multiple cancer types, with associated clinical metadata and drug response annotations. This wealth of data has enabled drug response modeling based on molecular profiles. Various machine learning methods have been proposed for drug response prediction in cancer. Jia et al. proposed a deep variational autoencoder for imputing drug response through compression of multiple genes into latent vectors in low dimensional space^12^. Ammad-Ud-Din, Muhammad, et al.^13^ reported a kernelized bayesian matrix factorization-based drug response prediction through incorporating prior knowledge of pathway-drug associations. Another approach utilized matrix factorization with similarity regularization for drug response prediction in cell lines by employing chemical structures of drugs and gene expression profiles^14^. Chang and colleagues proposed CDRscan, a convolutional neural net that leverages mutational signatures for predicting drug effectiveness^15^. Sakellaropoulos, Theodore, et al. reported gene expression data based deep neural network for drug response prediction that outperforms ElasticNet and Random Forest^16^.

By carefully surveying these methods, we identified two critical scopes for improvement. First, most of the past studies do not consider the structural properties of drugs as features (explanatory variables in predictive tasks). As a result, the machine learning models learn suboptimally and fail to make predictions on drugs that are not part of the training data. Second, gene expression levels are considered as independent variables, ignoring their pathway-specific combinatorial implication. Since most targeted therapies work through pathways, disregarding the pathway resolution causes overemphasis on machine learning techniques. Past studies have demonstrated the utility of using pathway enrichment scores for various downstream analyses, as opposed to gene expression values^17,18^. Notably, in our previous works, we have shown how pathway projection facilitates better modeling of biological processes^18,19^. As an added advantage, data integration based on pathway enrichment scores mitigates batch-effects^20^. While single-cell RNA-seq (scRNA-seq) enables the characterization of cellular heterogeneity in tumors, there is very little visible effort to leverage this fine-grained molecular information to predict drug response at sub-clonal resolution. This is primarily because most available training data, as indicated above, are bulk expression profiles, and training on bulk RNA-seq and testing on scRNA-seq is expected to give rise to misleading predictions. Pathway projections of scRNA-seq/bulk RNA-seq profiles reasonably alleviate this problem. Notable in this regard is the work by Suphavilai, Chayaporn, et al.,^21^ that describes a drug response prediction approach in head and neck cancer, leveraging scRNA-seq profiles. The authors, however, did not explore the utility of drug descriptors to generalize the prediction model.

In this work, we developed a deep neural network (DNN) based framework named Precily to predict drug response in both in vitro and in vivo settings. For model training, we made use of cell line-based high-throughput screening data (sources: CCLE, GDSC and CTRPv2). Convinced by the reproducibility and overall performance of the cell line model on unseen data (bulk and single-cell RNA-seq), we explored similar prediction tasks pertaining to in-house prostate cancer (PCa) cell lines and animal models under differential treatment conditions. As a proof of principle, we first evaluated Precily on differentially treated PCa cell lines. To ensure the cross-sample predictability of our model, we examined our LNCaP xenograft dataset mirroring in vitro treatments. For this, we utilized LNCaP xenografts from a PCa progression study where the tumors were harvested at different stages of treatment resistance. Precily predictions revealed clinically and biologically relevant associations of drugs and pathways in the context of treatment resistance and sensitivity. We also evaluated the utility of Precily in predicting response for drugs that had never been seen by the training models. For this, we considered metformin and orlistat, which are used for the treatment of type 2 diabetes^22^ and obesity^23^, respectively but also are found to have therapeutic potential in PCa. Finally, we used tumour RNA-seq data and the recorded clinical treatment response information from TCGA to validate the possibility of extrapolating our approach in precision oncology. We benchmarked the efficiency of the model, trained on patient samples from TCGA on RNA-seq profiles of pre-treatment, and matched post-relapse drug-resistant BRAF mutant melanoma patients. Our study connects a systematic drug response prediction pipeline with layered in vitro and in vivo comparisons involving cell lines, xenografts, and patient data, which is the most important prerequisite for the clinical implementation of such approaches.

## Results

### Precily enables reproducible drug response prediction in cancer cell lines

**Fig. 1:**
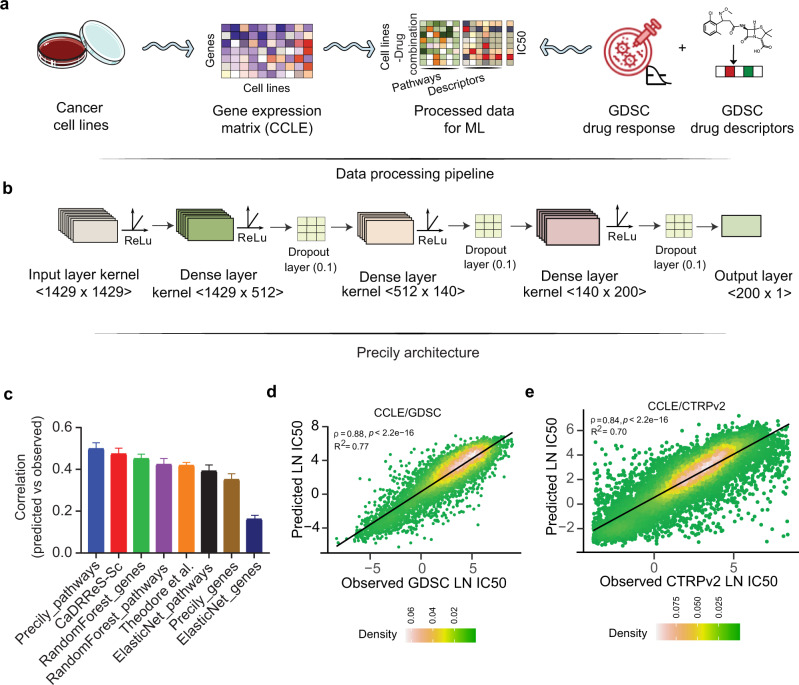
Illustration of the predictive analysis workflow of Precily. **a** Schematic workflow depicting the data processing pipeline of Precily. The first step involved the processing of training data. The RNA-seq gene expression (RSEM TPM) profiles from Cancer Cell Line Encyclopedia (CCLE) were subjected to pathway score transformation using GSVA. This GSVA score matrix was integrated with the drug descriptors obtained in the form of SMILES embedding for each compound. **b** Model architecture. The second step was the training of the ML model on this data, comprising GSVA scores and drug descriptors as an explanatory variable set and natural log-transformed IC50 values sourced from the GDSC database as the response variable. A deep neural network (DNN) from the Keras platform was used to perform the regression task of predicting drug response. **c** Comparison of drug response prediction across different approaches. Barplot shows the distribution of Pearson’s correlation coefficients for predicted vs. observed LN IC50 values for individual drugs (*n* = 173). Data are presented as mean values + /− SEM (Standard Error of the Mean). **d** Scatter plot demonstrating the performance of Precily across all cell line-drug pairs in the CCLE/GDSC test data. *P*-value was calculated using a two-sided *t*-test. **e** Scatter plot demonstrating the performance of Precily across all cell line-drug pairs in the CCLE/CTRPv2 test data. *P*-value was calculated using a two-sided *t*-test. Source data are provided in the Source Data file.

In this study, we present Precily, a deep neural network-based framework to model drug response using gene expression data in both in vitro and in vivo settings. For this, we leveraged bulk RNA-seq data of cancer cell lines from the Cancer Cell Line Encyclopedia (CCLE) for machine learning based prediction. For data preparation, first, for each of the 550 cancer cell lines CCLE, with available drug response data in GDSC, we computed pathway enrichment scores for 1329 canonical pathways from MSigDB^24^. Second, we obtained numeric molecular descriptors for 173 anti-cancer compounds (reported in GDSC) using SMILESVec^25^, by supplying simplified molecular-input line-entry system (SMILES) notation, retrieved using PubChemPy^26^. The summary statistics of the dataset used are provided in Supplementary Table 1. We found 550 cell lines common between both the databases screened against 173 unique molecular compounds for which SMILES notations were available. SMILESVec descriptors are vectors of size 100. We treated pathways and drug features as explanatory variables (*aka*. independent variables), while LN IC50 estimates as the decision variable (*aka*. dependent variable) under a regression framework. Both explanatory and decision variables are continuous in nature. The samples can be best understood as *(cell line, drug)* tuples with their responses. Here cell lines are encoded by pathway enrichment scores as discussed above (Fig. 1a). We used the open-source software Keras^27^ to build a DNN architecture comprising 2−6 hidden layers, tunable as a hyper-parameter (Fig. 1b). For model building, we followed cross-validation best practices and reported the performance on an independent test set. We realized that random train-validation-test splitting of *(cell line, drug)* tuples introduces data leak issues that do not mirror practical applications. In this way, training data becomes privy to cell line gene expression profiles and its sensitivity to some drugs, thereby making it rather easy to predict its sensitivity for a new drug. An analogous possibility is unlikely in clinical settings. Inferring drug responses in patients cannot be prejudiced upon accounts of past responses. In such a scenario, treatment naive cases cannot be tackled. To this end, we split the dataset based on the cell lines so that no cell line is common among the training, validation, and test sets. We compared our framework, named Precily, with two well-cited methods — Cancer Drug Response prediction using a Recommender System for single-cell RNA-seq (CaDRReS-Sc) by Suphavilai, Chayaporn, et al.^21^ and another method by Sakellaropoulos, Theodore, et al^16^. Both the methods utilize gene expression profiles for drug response prediction. We also considered traditional machine learning methods — random forest (RF) and ElasticNet, which have been used by previous studies for drug response prediction^28–30^. As a baseline, we evaluated the performance of RF, ElasticNet and Precily models using expression levels of 500 genes (as opposed to pathway score matrix), selected based on the squared coefficient of variation (CV^2^). On held out data, Precily based predictions attained the highest correlation with ground truth, closely followed by CaDRReS-Sc. Figure 1c shows distributions of Pearson’s correlation coefficients (ρ) across drugs, indicating the coherence between predictions by different methods and the ground truth LN IC50 values. The reason for presenting correlations at the level of drugs is that a major work^16^, we considered for comparisons, trained drug specific models using off-the-shelf H2O machine learning modules. In this way, one needs to manage one model for each compound. This approach is suboptimal since it does not leverage structural information of the compounds for prediction. For a global picture, we pooled our predictions across drugs and cell lines and obtained a Pearson’s correlation coefficient value of 0.88 (R^2^ = 0.77; *P*-value < 2.2e-16) (Fig. 1d).

While GDSC primarily catalogs anti-cancer drugs, the Cancer Therapeutics Response Portal v2 (CTRPv2) database features an assorted set of small molecules comprising tool compounds, probes and drugs, including US Food and Drug Administration (FDA)-approved cancer therapeutics^10^. We reciprocated a similar analysis of CCLE/GDSC on the CCLE/CTRPv2 combination. Notably, only 68 drugs were found common between GDSC2 and CTRPv2 datasets (Supplementary Fig. 1a). Further, we compared the distribution of LN IC50 values from GDSC2 and CTRPv2 datasets (Supplementary Fig. 1b**)**. Gross differences were observed, which prevented us from integrating the two. Precily yielded a Pearson’s correlation coefficient value of 0.84 (R^2^ = 0.70; *P*-value < 2.2e-16) (Fig. 1e), whereas CaDRReS-Sc obtained *ρ* = 0.83 (R^2^ = 0.68; *P*-value < 2.2e-16) (Supplementary Fig. 1c). Taken together, our analyses suggest that sensitivity to anti-cancer therapy can be predicted in cancer cell lines with reasonable accuracy and reproducibility.

### Drug response prediction leveraging single-cell expression profiles

**Fig. 2:**
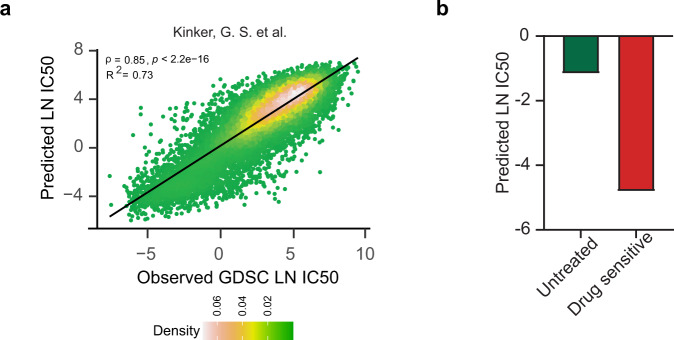
Assessment of Precily on scRNA-seq datasets. **a** Scatterplot demonstrating model efficiency on the Kinker, G. S. et al. scRNA-seq cell lines dataset comparing observed vs predicted LN IC50 measured by Pearson correlation (*ρ*) = 0.85 and coefficient of determination (R^2^) = 0.73. *P*-value was calculated using a two-sided *t*-test. **b** Evaluation of predicted drug response of paclitaxel in scRNA-seq MDA-MB-231 cells. Barplots depicting predicted response for paclitaxel in treatment-naive and paclitaxel sensitive cell population. Source data are provided in the Source Data file.

Single-cell RNA sequencing (scRNA-seq) technologies have refined our appreciation for intra and inter-tumoral heterogeneity across cancer types^19^. While scRNA-seq has been adopted as a method of choice for a large number of clinical studies, we are yet to fully exploit it to predict treatment outcomes at subclonal resolution by factoring in intra-tumor heterogeneity. To demonstrate the potential of Precily in predicting drug response at a single-cell level, we used single-cell datasets from two existing studies. First, we used scRNA-seq data produced by Kinker, G. S. and colleagues for 207 cancer cell lines, of which 116 cell lines overlap with the CCLE dataset^31^. We re-trained our CCLE/GDSC model such that the set of Kinker, G. S. et al. cell lines was never used in model training. We applied this model to the Kinker, G. S. et al. dataset and achieved a Pearson’s correlation coefficient=0.85 (R^2^ = 0.73; *P*-value<2.2e-16) (Fig. 2a). Further, we benchmarked our model using a second scRNA-seq dataset from a previously published study by Lee et al.^32^ consisting of treatment-naive metastatic breast cancer cells (MDA-MB-231) and a population of cells that had gained sensitivity to paclitaxel, after a drug-holiday period. In this study, the metastatic MDA-MB-231 cells were exposed to a paclitaxel drug. Most of the cells died after five days of exposure. However, some of the residual cells, cultured in drug-free medium after withdrawal of the drug, proliferated and established clones. Notably, these cells became more sensitive to paclitaxel on re-exposure. Precily trained on CCLE/GDSC data could correctly predict the in vitro therapeutic response from scRNA-seq data of the paclitaxel sensitive MDA-MB-231 cell population (Fig. 2b).

### Analysis of diverse treatment strategies in prostate cancer cell lines

**Fig. 3:**
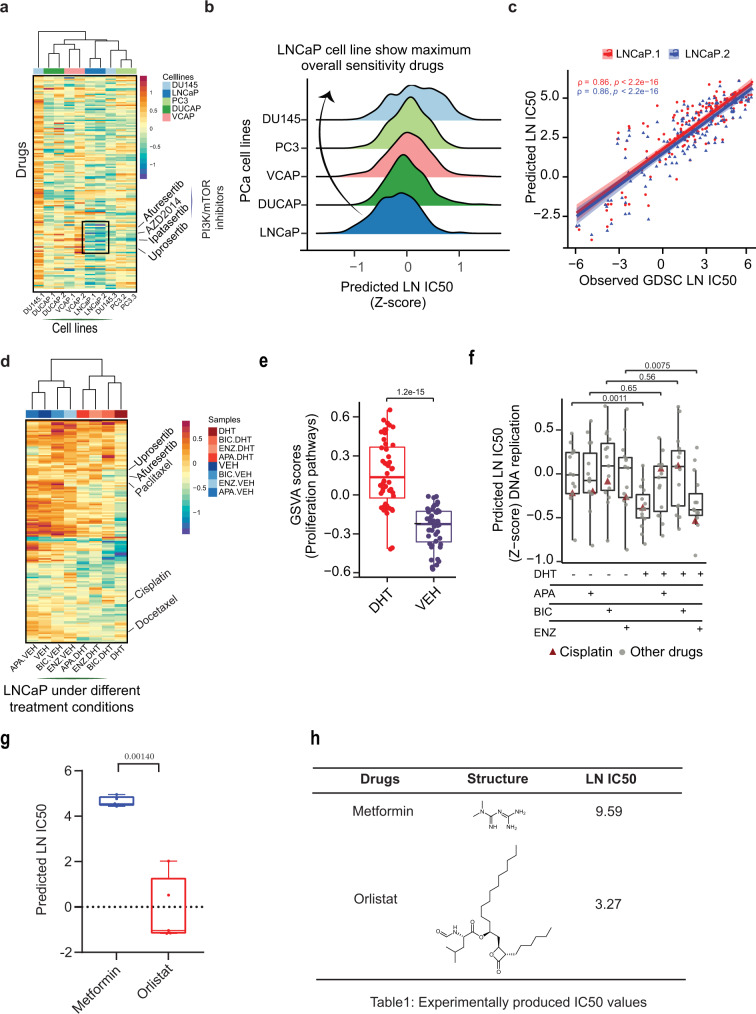
Analysis of drug response prediction in prostate cancer (PCa) cell lines. **a** Heatmap showing predicted LN IC50 (Z-score) for 155 drugs across five PCa baseline cell lines highlighting PI3K/mTOR signaling targeting drugs. The lower the LN IC50, the more sensitive a sample is predicted to a drug. Color bars indicate different cell lines. Euclidean distance was used for grouping cell lines. **b** Ridgeplot showing the overall distribution of predicted LN IC50 (Z-score) across five PCa cell lines, while pooling their predicted drug sensitivities across biological replicates. **c** Scatterplot depicting Pearson correlation (*ρ*) between observed and predicted LN IC50 for the LNCaP cell line. The line color indicated two biological replicates of the LNCaP cell line. *P*-value was calculated using a two-sided t-test. Shaded areas depict a 95% confidence interval. **d** Heatmap of predicted LN IC50 (Z-score) of LNCaP cells in the presence and absence of androgens (DHT) and AR antagonists (ENZ, BIC, and APA) to 155 drugs. Euclidean distance was used for grouping samples. **e** Boxplots depicting the distribution of GSVA scores of proliferation-related pathways in the presence and absence of DHT. Notably, *n* = 12 pathways and *n* = 48 samples originating from *n* = 8 treatment groups (DHT, BIC.DHT, ENZ.DHT, APA.DHT, VEH, BIC.VEH, ENZ.VEH and APA.VEH) have been considered for this analysis. *P*-values were obtained from the two-sided Wilcoxon rank-sum test. **f** Boxplot depicting predicted LN IC50 for DNA replication targeting drugs (*n* = 15) across all treatment conditions (*n* = 8). Cisplatin is denoted using darkred colored filled triangle and other drugs are represented using grey filled circles. *P*-values were obtained from the two-sided Wilcoxon rank-sum test. **g** Boxplot showing the distribution of predicted LN IC50 values by *n* = 5 pre-trained models (based on cross-validation and hyperparameter tuning) for two drugs, metformin and orlistat. *P-value* was obtained by using a two-sided *t*-test. As expected, the direction of the relative difference in drug sensitivity is captured correctly even at the log scale. The structure of these two drugs, along with observed IC50 values is also depicted in **h**. In all boxplots (**e**, **f**, **g**), the middle horizontal line represents the median value. Each box spans the lower quartile to the upper quartile. The whiskers indicate the minimum and maximum values within 1.5 times the IQR. Source data are provided in the Source Data file.

Despite therapeutic advances in PCa, treatment options remain limited, and the emergence of treatment resistance poses significant challenges. PCa is the most common malignancy in men and is curable if localized. Yet, for patients presenting with metastatic cancer, men are treated with androgen deprivation therapy (ADT) to exploit the unique dependence of PCa on androgen signaling for growth and progression. Although ADT is initially effective in most patients, the effect is temporary and cancer cells become resistant with the emergence of castration-resistant prostate cancer (CRPC). To which either taxanes or additional androgen targeted therapies such as enzalutamide are added with modest survival benefits, however, acquired resistance to these drugs eventually emerges. Thus, appropriate drug selection and combination remain crucial in the dynamically evolving landscape of cancer to derive maximum benefit for the patients^33–35^. Therefore, there is an unmet need for the selection of optimal drugs for PCa treatment^36,37^. We independently validated the CCLE/GDSC trained Precily model on our PCa datasets. We examined the concordance between our in-house gene expression data of PCa cell lines (i.e., LNCaP, DU145, PC3 and VCAP) with the same cell lines from the CCLE dataset and obtained a Pearson correlation in the range of 0.96 to 0.98 (Supplementary Fig. 2). Precily was applied on bulk RNA-seq profiles of five untreated PCa cell lines, each with two biological repeats. We predicted drug responses for each of these ten samples for 155 drugs tested against PCa cell lines in the GDSC database targeting various cellular pathways. Androgen Receptor (AR) positive PCa cell lines (LNCaP, DUCAP, and VCAP) were predicted to be relatively more sensitive to the drugs as compared to AR negative cell lines (DU145 and PC3). The median Z-scores associated with predicted LN IC50 values (across GDSC drugs) for LNCaP, DUCAP, VCAP, DU145, and PC3 were recorded as −0.17, −0.03, 0.02, 0.17 and 0.06 respectively (Fig. 3a, b). Of these five cell lines, LNCaP cells were predicted to be the most sensitive to these drugs (Fig. 3b). Precily predictions clearly highlighted the potential sensitivity of LNCaP cells to PI3K/mTOR signaling pathway targeting drugs such as broad spectrum of AKT inhibitors, ipatasertib, afuresertib, and uprosertib, and in particular, the mTORC inhibitor AZD2014 (Supplementary Fig. 3a, b). We noted elevated GSVA pathway scores for mTOR-related signaling among LNCaP gene expression profiles, which may explain the predicted sensitivity to these drugs (Supplementary Fig. 3). For this analysis, we removed the concerned cell lines under testing from the CCLE/GDSC training data. When comparing the predicted LN IC50 values with the corresponding GDSC values across drugs, we observed a Pearson correlation of 0.86 (two-sided *t*-test *P*-value < 2.2e - 16) for the two biological replicates of LNCaP cells, respectively (Fig. 3c).

We were further interested in testing how the drug response prediction was altered when LNCaP cells were cultured in the presence of the androgen receptor (AR) agonist dihydrotestosterone (DHT) as compared to the vehicle control (VEH) in androgen deprived media conditions; and furthermore, how treatment with the clinically approved AR antagonists bicalutamide (BIC), enzalutamide (ENZ), and apalutamide (APA) under these conditions affected the predicted sensitivity pattern. Predicted sensitivity of a broad spectrum of anti-cancer drugs on treated samples may represent putative altered phenotypic landscapes, indicating therapeutic consequences. In clinical settings, evaluation of post-treatment sensitivity patterns may be crucial for identifying pairing therapies to combat the diverse spectrum of PCa resistance. Overall, LNCaP cells cultured with DHT were predicted to be more sensitive to therapeutic drugs as compared to cells cultured without DHT and AR antagonists (Fig. 3d, Supplementary Fig. 4a). The cells cultured in the presence of DHT were observed to have elevated GSVA scores for the proliferation-associated pathways, which would be expected as DHT is known to stimulate PCa cell proliferation^38,39^. This supports the notion that actively proliferating cells are more sensitive to specific anti-cancer drugs, while cells in a cytostatic or quiescent state are more resistant (Fig. 3e). Notably, the addition of AR antagonists in the presence of DHT did not fully reverse the predicted DHT conferred drug sensitivity. In fact, our model predicts that even in the presence of ENZ, cells remain sensitive toward cisplatin, docetaxel and paclitaxel, the latter two mainstay chemotherapeutic drugs currently in clinical use for advanced PCa patients (Fig. 3f, Supplementary Fig. 4b). In contrast, the presence of ENZ is predicted to decrease sensitivity towards a subset of drugs, for example, the PI3K/mTOR pathway inhibitors uprosertib and afuresertib (Supplementary Fig. 4c). These findings allude to the use of Precily in identifying potential combinatorial therapies. An exciting application of our approach could be predicting response to unseen compounds that are not part of the training data. To demonstrate the same, we considered metformin and orlistat, two drugs that are primarily used for type 2 diabetes and obesity, respectively; however, a rising number of reports suggest their therapeutic potential in some cancers. Precily based prediction of the sensitivity of LNCaP cell line to these drugs was found to be consistent at a relative scale (Fig. 3g, h).

### Precily predictions in xenografts concur with broad mechanistic reasoning

**Fig. 4:**
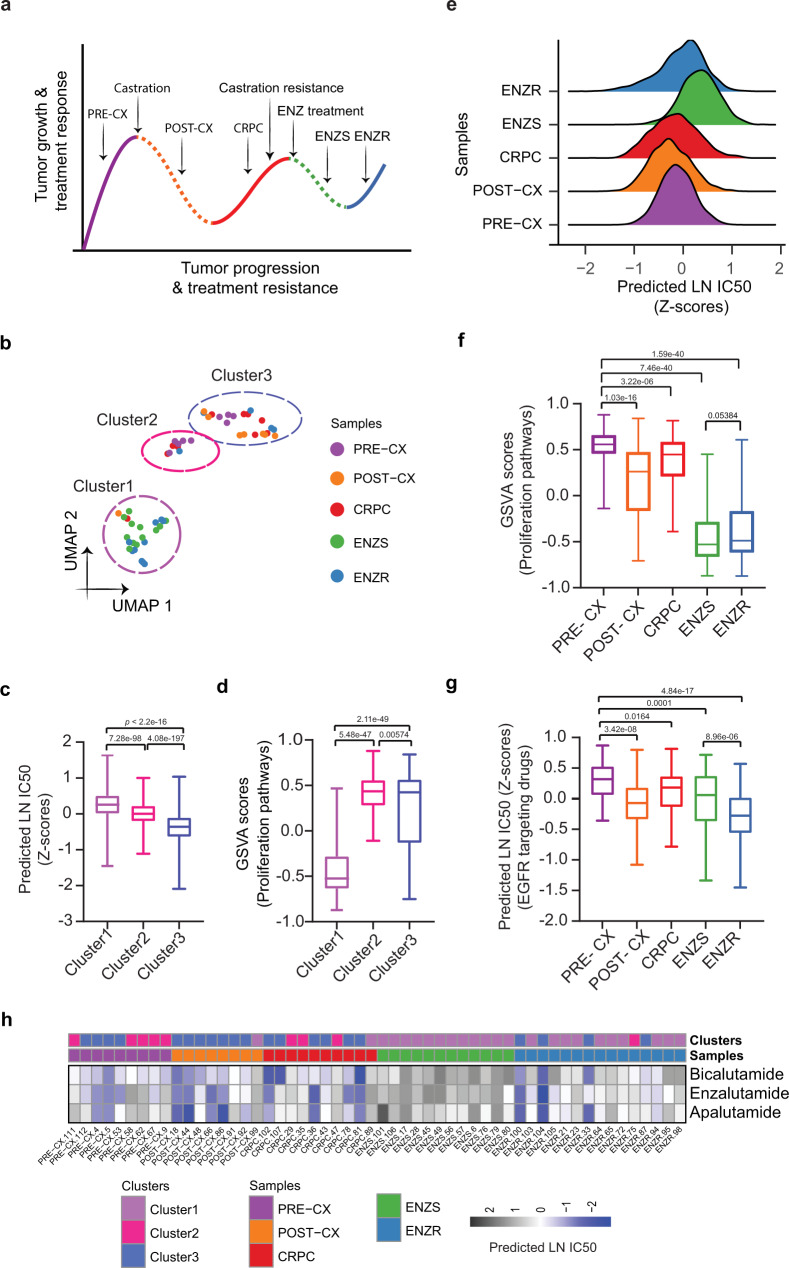
Analysis of drug response prediction in LNCaP derived xenografts. **a** Overall schematics of the experimental setup of LNCaP xenograft-based PCa progression study with indicated treatments, therapeutic response, and therapeutic resistance stages. Solid lines represent growth and treatment resistance; dotted lines represent treatment responsiveness. **b** Uniform Manifold Approximation and Projection **(**UMAP) based projections of predicted LN IC50 showing three separate clusters. We subjected predictions to principal component analysis (PCA) and used the first 10 principal components as input for UMAP based embedding. **c** Boxplots depicting the distribution of predicted LN IC50 (Z-score) across three clusters. Each data point relates to a xenograft tumor sample-drug pair, wherein a sample belongs to one of the clusters as shown in Fig. 4b (*n* = 24, *n* = 9 and *n* = 21 samples from cluster 1, cluster 2 and cluster 3, respectively) and a GDSC drug (*n* = 155). *P*-values were obtained from two-sided Wilcoxon rank-sum test. **d** Boxplots showing the distribution of GSVA scores of proliferation-related pathways (*n* = 12) across three clusters (*n* = 24, *n* = 9 and *n* = 21 samples from cluster 1, cluster 2 and cluster 3, respectively). P-values were obtained from the two-sided Wilcoxon rank-sum test. **e** Ridgeplot showing the overall distribution of predicted LN IC50 (Z-score) across tumor types. **f** Boxplots showing the distribution of GSVA scores of proliferation-related pathways (*n* = 12) across tumor types (*n* = 9 PRE-CX, *n* = 8 POST-CX, *n* = 10 CRPC, *n* = 12 ENZS and *n* = 15 ENZR). *P*-values were obtained from the two-sided Wilcoxon rank-sum test. **g** Box plot depicting predicted LN IC50 (Z-score) of EGFR signaling pathway targeting drugs. We examined *n* = 7 drugs against 54 tumor bulk RNA-seq samples (*n* = 9 PRE-CX, *n* = 8 POST-CX, *n* = 10 CRPC, *n* = 12 ENZS and *n* = 15 ENZR), in every possible combination. *P*-values were obtained from a two-sided Wilcoxon rank-sum test. **h** Heatmap showing predicted LN IC50 for three unseen drugs not present in GDSC (BIC, APA, and ENZ). Color bars indicate tumor types and clusters as acquired through UMAP projections. In all boxplots (**c**, **d**, **f**, **g**), the middle horizontal line represents the median value. Each box spans the lower quartile to the upper quartile. The whiskers indicate the minimum and maximum values within 1.5 times the IQR. Source data are provided in the Source Data file.

Xenografts are useful in vivo tumor models for directly investigating therapeutic response and predicting anti-cancer drug response in patients with cancer of a similar phenotype. As such, we evaluated our ability to predict drug response in cell line derived xenografts. We used our bulk RNA-seq data from LNCaP xenografts derived from a large and well-annotated PCa progression study investigating responsiveness and subsequent resistance to therapies targeting the AR. LNCaP xenograft tumor establishment and initial growth are dependent on androgens in male mice (PRE-CX). Upon castration, AR activity and tumor growth are suppressed (POST-CX), however, this initial responsiveness to castration reproducibly gives way to castration-resistance (CRPC). Further treatment of CRPC with ENZ initially provides a therapeutic response (ENZ Sensitive; ENZS), however, resistance emerges with time (ENZ Resistant; ENZR) (Fig. 4a). Using CCLE/GDSC trained Precily model, we predicted the drug response for each of the 54 samples across this spectrum of successive therapeutic responsive and resistance states. The LNCaP xenograft tumor samples clustered into three main groups based on their overall predicted sensitivity to the 155 drugs (tested in the GDSC study against PCa cell lines) in the analysis (Fig. 4b, Supplementary Fig. 5a). Cluster 1 samples had the most resistant tumors, which correlated to their lower proliferative index. Cluster 1 predominantly consisted of ENZ-treated tumor samples (10 of the total 15 ENZR and all 12 ENZS samples). In contrast, cluster 3 samples had the highest predicted overall sensitivity to the 155 drugs, which may be attributed to their higher proliferative index, indicated by higher GSVA pathway scores for cell proliferation-associated gene sets (Fig. 4c, d). ENZR tumors were distributed across all three clusters, thereby indicating heterogeneous outcomes of the treatment. We hypothesize that ENZ resistance is acquired through different underlying mechanisms and may be inflicted with the contribution of stromal components in the tumor microenvironments. The indication of multiple ENZ resistance mechanisms was strengthened by the multimodal distribution of predicted LN IC50 for ENZR tumors compared to the uniform distribution in the case of ENZS tumors **(**Fig. 4e). In contrast to ENZS, the most resistant sample type, ENZR samples were predicted to develop (regain) some level of sensitivity to a subset of drugs. ENZR samples tended to have higher GSVA scores of proliferation-associated pathways relative to ENZS samples, however, this did not reach statistical significance (Fig. 4f). ENZR tumors were predicted to be more sensitive to EGFR targeting drugs than any other tumor type in the study (Fig. 4g), with sapitinib having the most profound effect (Supplementary Fig. 5b). While we achieved encouraging results on the drugs within our training set, we could also predict biologically relevant responses for drugs not included in the training set— APA, BIC, and ENZ. We observed sensitivity to AR antagonists in the PRE-CX, POST-CX and CRPC groups. However, for ENZ treated ENZS and ENZR groups, our model predicted a decline of sensitivity. Our analyses suggest that responsive ENZS tumors from mice actively treated with ENZ are unlikely to benefit from additional AR antagonists **(**Fig. 4h**)**.

### Predictability of clinical response in patients

**Fig. 5:**
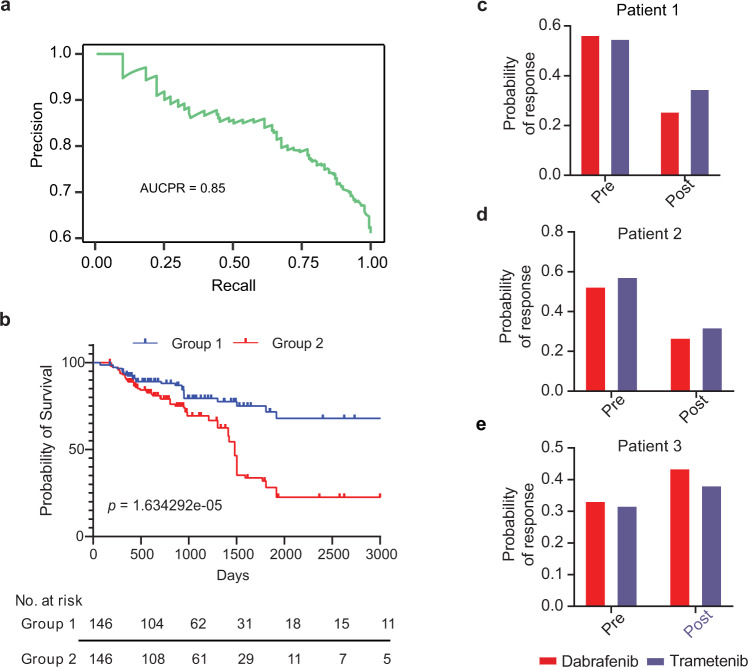
Evaluation of TCGA model efficiency. **a** Precision-recall curve representing the performance of the best AutoML model on the TCGA test dataset. **b** Survival analysis of a test dataset spanning multiple TCGA cancer types. Patients were classified into two groups based on the median value of the predicted probability of response with a *P-value* = 1.634292e-05 (log-rank test). We estimated the 5-years survival probability of group 1 as 0.72 and group 2 as 0.28. **c–e** Bar plots showing the probability of predicted response for drugs dabrafenib and trametinib in patient 1, patient 2, and patient 3, respectively. A red bar indicates the probability of response for dabrafenib and a purple bar indicates the probability of response for trametinib. Source data are provided in the Source Data file.

The Cancer Genome Atlas (TCGA) features a large compendium of omic datasets spanning multiple cancer types with gene expression profiles of mostly primary patient tumors and clinical response information. The clinical drug response data includes patient demographics and responses to administered drugs. While sticking to the Precily approach, we aimed to model complete/partial versus non-response in TCGA patients. After filtering and preprocessing, we were left with 3108 patient-drug combinations with recorded clinical responses. These entailed 1443 unique patients (multiple drugs were administered to some patients) and 139 unique drugs, representing 29 cancer types. Given the bewildering diversity of cancer genomes and compound structures, these numbers are clearly inadequate. We, therefore, performed a pooled analysis of the data, agnostic of cancer types/stages. Our formulation of the modeling task allows learning and prediction on virtually any combination of sample-drug pairs. The data matrix prepared for machine learning featured 1427 explanatory variables (1327 pathways plus 100-sized drug descriptor vectors). Due to the data paucity, unlike cell lines, in this case, we used AutoML, the off-the-shelf R library by H2O.ai (https://docs.h2o.ai/h2o/latest-stable/h2o-docs/automl.html)^40^ to build a drug response classifier on the basis of tumor bulk RNA-seq data from TCGA. The summary statistics of the dataset used are provided as supplementary data (Supplementary Table 2). 90% of the complete data (3108×1427 dimensional matrix) were used for 5-fold cross-validation and hyperparameter tuning, while the remaining 10% were held out for independent testing. AutoML evaluated a total of 34 models (including machine learning, deep learning, boosted, and ensemble models) and offered ‘Extremely Randomized Trees’ (XRT) as the best model (Supplementary Data 1). Our train-validation-test split ensured there were no overlapping patients in the datasets. XRT obtained an AUC-PR of 0.85 on the test data (Fig. 5a). We tested if the incorporation of cancer stage information improves drug response predictions. To our surprise, its inclusion disadvantaged the model performance (AUC-PR = 0.79), suggesting a lack of objectivity in cancer staging. Notably, among the 34 tested classifiers, the best variant of the deep neural network ranked 19th. This can be explained by the paucity of patient data. As expected, a classifier variant of the Precily DNN architecture yielded a suboptimal AUC-PR of 0.77. Independently, we evaluated if drug response probability can be used as a yardstick for survival risk stratification. Significant improvement in overall survival was observed in patients administered with drugs that the model predicted to be effective (Fig. 5b). We used the median of response probability as a cut-off to create two groups for the survival analysis. As an independent evaluation, we assessed the association of Precily predictions with patient survival, in the presence of other common covariates (cancer type and stage). A multivariate cox regression analysis yielded a Likelihood ratio test *P-value* =*<*  *2.2e-16*. The covariate Precily predicted probability of response yielded a *P-value* = 0.00135 (Wald test) indicating its independent association with survival. Worth highlighting the sparse availability of data at the resolution of cancer/drug/stage, which makes it difficult to gauge the clinical applicability of our patient model (Supplementary Fig. 6a, b**)**. Group wise contingency tables comparing predicted and ground truth responses are shown in Supplementary Fig. 6c, d. All three BRCA tumour-drug pairs (TCGA-A8-A08O-Anastrozole; TCGA-A8-A08O-Vinorelbine; TCGA-Z7-A8R5-Paclitaxel) that were mapped to Group 2 (poor survival), were marked non-responders in TCGA. TCGA-A8-A08O and TCGA-Z7-A8R5 were stage IV and stage IIIA patients, respectively. Out of 49 tumor-drug pairs, only seven were non-responders, including these three pairs. Notably, there were many stage III patients in Group 1, of which most were responsive to the treatment (Supplementary Data 2). One can tune the probability cut-off to strike the desired balance between sensitivity and specificity.

Next, we applied the TCGA data model to an independent dataset for which pre and post-treatment RNA-seq profiles and clinical response information were available^41^. We predicted the drug response of RAF inhibitor (dabrafenib) and MEK inhibitor (trametinib) in three pre-treatment and matched post-relapse BRAF-mutant RAF/MEK inhibitor-resistant melanoma patients. The journeys of three patients are documented in supplementary data (Supplementary Note 1). Wagle, Nikhil, et al. reported the presence of MEK2^Q60P^ mutation, BRAF Splice Isoform, and BRAF amplification in the post-treatment patient 1, patient 2, and patient 3, respectively, as revealed through whole-exome sequencing (WES) and RNA-seq. These alterations may be a potential cause of conferring resistance to RAF/MEK inhibitors in these patients^41^. Notably, our prediction results revealed a similar trend, whereby our probability of response to dabrafenib and trametinib in pre-treatment patient 1 and patient 2 was higher than post-treatment (Fig. 5c, d). Furthermore, we were correctly able to predict these patients as responders. This aligns with the original annotations of the study as these patients are categorized as a partial response based on Response Evaluation Criteria In Solid Tumors (RECIST)^42^. In contrast, patient 3 was correctly predicted to be resistant to dabrafenib for both pre and post-treatment samples **(**Fig. 5e**)**. This is concordant with the original study where this patient was categorized as stable disease based on RECIST criteria.

## Discussion

Predicting the drug response of cancer cells is of paramount importance in personalized oncology. In this study, we developed Precily, a deep neural network (DNN) based framework to predict the response to cancer therapy based on gene expression profiles and drug descriptors. Due to the explicit use of pathway enrichment scores, our model highlights the underpinning biological mechanisms contributing to drug resistance. Furthermore, the pathway-based prediction approach allowed us to infer cellular fates upon treatment from single-cell expression data accurately. This can enable drug response prediction at sub-clonal resolution using tumor scRNA-seq data.

We evaluated Precily predictions by showing a reasonable correlation between the experimental LN IC50 for LNCaP cells used in the training data set and the model-predicted values. Further, we noted that the *PTEN* negative LNCaP cell line was predicted to have an elevated sensitivity to PI3K/mTOR signaling targeting drugs. Built on pathway scores, Precily allows exploring the association between pathway activities and drug response. This might help identify unexplored signaling pathways as potential therapeutic targets in PCa. Notably, previous studies have broadly suggested that increased PI3K/AKT/mTOR signaling is associated with sensitivity to PI3K inhibitors in LNCaP cell lines and other *PTEN* null cancer cell lines. Furthermore, mTOR inhibitors might be effective against *PTEN* null tumors^43^.

We further explored how drug sensitivity prediction indicated a switch of cellular states into drug-responsive and resistant states in LNCaP cells and xenografts. In the presence of androgens, LNCaP cells were predicted to be more sensitive to cancer therapeutics that target highly proliferative cells. This is expected as androgens drive proliferation in AR-positive PCa cell lines and xenografts^38,39^. AR antagonists are the primary treatment option for metastatic PCa, which antagonize cellular androgen response pathways on a molecular level. With AR antagonist treatments, LNCaP cells were predicted to have pronounced similarities and differences for ENZ, APA, and BIC treatments, mirroring the complex biological underpinning of the treatment responses. A strong reversal by an AR antagonist of DHT conferred sensitivity was observed for drugs targeting the PI3K/mTOR pathway, with ENZ showing the most profound effect. In contrast, ENZ treatment was predicted to further increase the DHT conferred sensitivity to selected drugs, including cisplatin, docetaxel and paclitaxel, while other AR antagonists may not, e.g. BIC and APA for cisplatin. These predictions suggest that patients on active treatment with ENZ may still benefit from added chemotherapy using cisplatin, while patients on treatment with BIC or APA may not. These predictable differential effects are important considerations for combinatorial therapy to maximize the therapeutic benefit for a given patient while minimizing detrimental side effects of chemotherapy affecting the quality of life.

To further evaluate the applicability of the drug sensitivity predictions with Precily, we used data derived from our well-annotated PCa xenograft model, following the progression from early androgen-responsive to CRPC, and then to ENZ treatment responsive and ENZ resistant states. The sensitivity predictions highlight changing vulnerabilities of the tumors in different stages of progression and treatment. Notably, we observed that ENZR tumors are predicted to develop a susceptibility to selected therapeutics providing a new window of opportunity for therapeutic strategies. For example, our model predicted sensitivity to specific drugs targeting the EGFR signaling pathway in case of ENZ treatment, highlighting sapitinib as a potential therapeutic for ENZ resistant patients. While prostate tumors in an androgen replete setting are predicted to be highly resistant to EGFR targeting drugs, a distinct vulnerability is developed during progression to ENZR. As these drugs targeting the EGFR pathway are approved for cancer treatment in other cancer types, this may facilitate the clinical evaluation of combination therapy in PCa patients treated with ENZ or those that have developed ENZR. Previous studies have suggested that a combination of ENZ and EGFR targeting inhibitors might be an effective therapeutic strategy in overcoming ENZ resistance^44^. Further laboratory and preclinical studies into the molecular mechanism behind our predicted sensitivity pattern are needed to confirm these findings.

Our evaluation of the TCGA model using an external BRAF-mutant melanoma dataset resulted in clinically relevant predictions based on tumor RNA-seq samples from the patients. The probability of response for the patients classified in the partial response category was higher than post-treatment due to acquired resistance to the first line of therapy. This suggests that top drugs predicted by our method, for example, cyclophosphamide and cisplatin, might serve as alternative therapies in combination with other drugs to overcome acquired resistance. This warrants further investigation.

The major advantage of the discussed framework is that the associated models can be used to infer drug sensitivity for virtually any sample-drug pairs due to the use of numeric drug descriptors. First, this approach enables us to pool cell-drug combinations across cancers, thus providing an opportunity to improve the model performance. Second, the successful prediction by Precily of LNCaP sensitivity to metformin and orlistat confirmed that Precily could be potentially used to evaluate the efficacy of drugs that are not part of the modeling task. Third, Precily based monotherapy sensitivity predictions can provide cues for clinically plausible combination therapies. Thus, Precily can be employed as a first pass screening tool to aid in clinical decisions.

A limitation of Precily is that at the level of individual drugs, correlations between observed and predicted IC50 values were suboptimal. However, between drugs, the relative sensitivities are captured reasonably well. We obtained promising results while testing the approach on patient tumor data. However, there is a scarcity of human cancer data with recorded clinical drug responses. With limited data of assorted cancer types from TCGA, we developed and validated a pan-cancer model for drug response prediction, which we believe can be further substantiated with additional data curated from diverse clinical trial studies.

To summarize, our current work links bioinformatic predictions of drug response to clinically explainable observations, both in in vitro and in vivo settings. Given only a limited number of clinically relevant molecular subtypes are known in case of PCa, drug response inference based on tumor bulk expression profiles may be leveraged in furthering pharmaco-genomic research.

## Methods

### Ethical approval

Animal studies were performed with the approval of the University of Queensland and Queensland University of Technology (QUT) Animal Ethics Committees (ethics approval number QUT/572/17) and in accordance with accepted standards of humane animal care as outlined in the ‘Australian Code of Practice for the Care and Use of Animals for Scientific Purposes’ and the universities’ guidelines for the use of laboratory animals.

### In vitro prostate cancer (PCa) cell line experiments

The human prostate cancer cell lines LNCaP (CVCL_0395, ATCC #CRL-1740 clone FGC), VCaP (CVCL_2235, ATCC #CRL-2876), DU145 (CVCL_0105, ATCC #HTB-81), PC3 (CVCL_0035, ATCC #CRL-1435) and DUCAP (CVCL_2025, generous gift from Dr. Matthias Ness, VTT Technical Research Centre of Finland) were cultured in Phenol-red free RPMI medium-1640 (Thermo Fisher Scientific/ Life Technologies, #11835) supplemented with 5% fetal bovine serum (FBS, Sigma #F2442) in a humidified incubator at 37 °C and 5% CO2 and untreated samples were harvested for RNA extraction during their exponential growth phase. Cell lines were authenticated using STR (short tandem repeat) analysis as described in the ANSI Standard (ASN-0002) 2012 by the ATCC Standards Development Organization. Nine short tandem repeat (STR) loci plus the gender determining locus, Amelogenin, were amplified using the commercially available GenePrint®10 System kit from Promega. The cell line samples were processed using the Applied Biosystems® 3500 Genetic Analyzer. Data were analyzed using GeneMapper® v5.0 software (Applied Biosystems). Appropriate positive and negative controls were run and confirmed for each sample submitted. STR profiles of query samples are compared to the ATCC STR Database (or similar) to verify cell line identity. Cell lines with ≥80% match are considered to be related i.e., derived from a common ancestry. For regular mycoplasma testing in cell cultures, the MycoAlert® Mycoplasma Detection Kit (Lonza, #LT07-318), a sensitive luciferase-based biochemical test that detects the activity of mycoplasma enzymes, is used according to manufacturer’s instructions. All cell lines were checked against the list of known misidentified cell lines maintained by the International Cell Line Authentication Committee (ICLAC).

For in vitro treatment experiments, LNCaP cells were seeded and cultured for 72 h in a standard medium with 5% FBS. This was followed by a 48 h incubation in androgen-depleted conditions using medium +5% charcoal-stripped serum (CSS, Sigma #F6765). Treatment with the androgen targeting drugs (ATTs) enzalutamide (MDV3100, Selleck Chemicals, #S1250) (10 uM), bicalutamide (Selleck Chemicals, #S1190) (10 uM), and apalutamide (Selleck Chemicals, #S2840) (10 uM) was performed for 48 h, either in the absence or presence of 10 nM dihydrotestosterone (DHT, dissolved in EtOH).

LNCaP cells were seeded for 24 h into 96-well tissue culture plates (Corning #COR3599) and treated with serial dilutions of the indicated compounds. Cell viability as a function of metabolic activity was measured by an AlamarBlue (Thermo Fisher Scientific #DAL1025) endpoint assay according to the manufacturer’s instructions (fluorescence measured at 560/590 nm excitation/emission)^45^. Calculations of half-maximal inhibitory concentration (IC50) after treatment with the respective drugs were performed with GraphPad Prism. Each data point was performed in triplicate and repeated in at least three independent experiments.

### Establishment of LNCaP xenografts

Five to six-week-old male NOD-SCID mice (Nonobese diabetic/severe combined immunodeficiency, mutant inbred, NOD.CB17-Prkdc^scid^/Arc) were sourced from the ‘Animal Resource Centre’ (Murdoch, Western Australia) and underwent a minimum one week acclimatization period upon arrival. Mice were group housed in individually ventilated cages at a 12 h light/dark cycle under specific pathogen-free, temperature- (22–24 degrees Celsius) and humidity-controlled (50–65%) conditions, fed standard chow (Specialty Feeds, Australia) and water ad libitum. For the in vivo tumor progression study, xenografts were established by subcutaneous injection of one million LNCaP cells (CVCL_0395, ATCC #CRL-1740 clone FGC) into the flank of anaesthetised mice (54 in total; isoflurane inhaled anaesthetic in oxygen). Tumor volume was measured thrice weekly using digital callipers; weight and clinical health score tracked thrice weekly and daily, respectively. Submandibular bleeds were undertaken weekly to collect blood (~100 ul) which was used to track serum PSA (Prostate-Specific Antigen). At a tumor size of ~200 cubic mm (mm^3^), mice were either surgically castrated (45 mice) or received mock surgery for the PRE-CX group (9 mice) under aseptic conditions, whilst anaesthetised and provided analgesic (carprofen). Tumors from the PRE-CX group (9 mice) were harvested when the ethical tumor size endpoint of 1000 mm^3^ was reached. Tumors from the POST-CX (post castration, 8 mice) group were harvested one-week post-castration at primary serum PSA nadir (GenWay-Biotech ELISA, as per manufacturer’s instructions). CRPC (Castrate-Resistant Prostate Cancer, 10 mice) tumors were harvested when PSA levels recurred to pre-castration levels or when tumor burden reached 1000 mm^3^ post-castration. For the enzalutamide (ENZ) groups, treatment with 10 mg/kg ENZ (oral gavage, 5 days/week) commenced as serum PSA began to rise post-castration. Tumors were harvested either at secondary PSA nadir (ENZS, 12 mice) while on ENZ treatment, with the remaining tumours collected when PSA had recurred to pre-ENZ treatment levels or when tumor volume reached ethical endpoint of 1000 mm^3^ despite ENZ treatment (ENZR, 15 mice). Xenografts were rapidly dissected, snap frozen in liquid nitrogen and stored at −80 °C; until being homogenised in a pre-cooled TissueLyser (Qiagen) for RNA extraction.

### RNA extraction, library preparation, and bulk RNA-sequencing

For mRNAseq, total cellular RNA was extracted using the Norgen RNA Purification PLUS kit #48400 (Norgen Biotek Corp., Thorold, Canada) according to the manufacturer’s instructions, including DNase treatment. RNA quality and quantity were determined on an Agilent 2100 Bioanalyzer (Agilent Technologies, Santa Clara, USA) and Qubit®. 2.0 Fluorometer (Thermo Fisher Scientific Inc, Waltham, USA). Library preparation and sequencing was performed using the Illumina TruSeq Stranded mRNA Sample Prep Kit (strand-specific, polyA enriched, Illumina, San Diego, USA) with an input of 500 ng − 1 ug total RNA (RIN > 8), followed by paired-end sequencing with a read length of 100–150 bp and yielding about 30–60 M read pairs per sample.

RNAseq raw data was processed through a custom-designed pipeline. Raw reads were assessed with FastQC^46^, then trimmed using TrimGalore^47^, followed by alignments to the human genome (GRCh38 / hg38) and transcriptome (Ensembl.v.99 / Gencode.v.33, Jan-2020) using the STAR^48^ aligner and read quantification with RSEM^49^. For xenograft samples, STAR alignment was performed against a chimeric human+mouse reference (mouse: GRCm38 / mm10, Gencode.v.M24 / Ensembl.v.99, Jan-2020), followed by RSEM read quantification. Only reads aligned to the human genome were used for downstream analysis. TPM values from the RSEM output were used for GSVA scoring. For predictions, we used averaged GSVA scores of the biological replicates of LNCaP cell line under different treatment conditions.

### Gene expression data of cancer cell lines

For machine learning based modeling of drug response measured by half-maximal inhibitory concentration (LN IC50), we used publicly available TPM (transcript per million) normalized RNA-seq gene expression profiles of 1019 Cancer Cell Line Encyclopedia (CCLE)^8^ cell lines quantified using the RSEM (RNA-Seq by Expectation-Maximization) software. The corresponding drug response information for the cell lines was sourced from the GDSC2 dataset of the Genomics of Drug Sensitivity in Cancer (GDSC) database^9^. In the GDSC2 dataset, some drug-cell line pairs have multiple LN IC50 measurements. In such cases, we averaged the LN IC50 values. The RNA-seq gene expression profiles of 550 CCLE cell lines overlapping with the GDSC2 dataset cell lines were used for the model training. This matrix contained 57820 Ensembl Gene IDs, which were converted into official gene symbols using gencode.v19.genes.v7_model.patched_contigs.gtf annotation file. This resulted in multiple Ensembl gene IDs corresponding to some of the individual gene symbols. We considered the average expression in such cases. At this stage, our expression matrix comprised 54301 genes and 550 cell lines. This matrix was subjected to log2 transformation with the addition of a pseudo count of 1.

### Tumor RNA-seq data

Analogous to cell lines, on tumor mRNA sequencing data from TCGA, we modeled drug-response in terms of responder and non-responder. TCGA RNA-seq data were downloaded from the Broad GDAC firehose^50^ encompassing 33 tumor types. We used Illumina HiSeq RNA-seq v2 data processed at the gene level using RSEM^49^. The clinical drug response information for the patient samples was fetched from the NCI Genomic Data Commons portal^51^. Drug names and response information was collected from clinical metadata and rectified for typographical and spelling errors and to harmonize commercial names and molecular drug names. We categorized complete response and partial response patients as responders. Patients with clinically progressive and stable diseases were marked as non-responders. RNA-seq gene expression profiles of cancer types with clinical response data for fewer than two patients were excluded. At this stage, the filtered data contained gene expression profiles for 29 cancer types. Then for individual cancer types, scaled estimates from gene-level RSEM files were transformed into TPM by multiplying with a factor of 1e6 followed by log2 transformation with the addition of pseudo count 1. For the patients with identical TCGA barcodes, we averaged their gene expression profiles for downstream analysis.

### Drug descriptor data

We obtained drug response information for 192 compounds from the GDSC2 dataset for the 550 cell lines in the CCLE dataset and clinical response information for 215 compounds for 1517 TCGA patients. The chemical structure information for these molecular compounds was retrieved in terms of a simplified molecular-input line-entry system (SMILES) using PubChemPy^26^. However, SMILES were not available for all the molecular compounds. As a result, we ended up with SMILES of 173 and 139 compounds for 550 CCLE cell lines and 1443 unique TCGA patients, respectively. The SMILESVec python tool was used to convert these SMILES into vector embeddings by utilizing data of embeddings trained on Pubchem and embedding of size 100 ^25^.

### Pathway activity scores

To train models, we used pathway activity scores. We used the Gene Set Variation Analysis (GSVA)^17^ R software package to compute GSVA scores based on the log_2_(TPM  +  1) gene expression matrix for selected gene sets from Molecular Signatures Database (MSigDB)^24^ with min.sz set as 5. We used the c2 collection of canonical pathways (MSigDB.CP.v.6.1) consisting of 1329 gene sets. We integrated the pathway score matrix with the vector embeddings of the drug features. Our final CCLE cell line training dataset constituted 80056 cell line-drug combinations in rows and 1429 features entailing 1329 pathways and drug features of vector size 100 for each molecular compound as the explanatory variable and LN IC50 as the response variable in columns. For TCGA patient data, gene expression profiles of individual cancer types were transformed into pathway scores. The GSVA scores of the samples where drug response information was available in each cancer category were merged based on common pathways. The final matrix consisted of 3108 patient-drug combinations and, 1427 features (pathways & drug descriptors) and the response variable (responder = 1, non-responder = 0).

### Training models using CCLE RNA-seq cell line dataset

We formulated the drug response prediction task with genes/pathway enrichment scores, in tandem with drug descriptors as a regression problem. We utilized various machine learning techniques for response prediction. We split the CCLE training dataset into a 90% training set (72262 cell line-drug pairs) and a 10% test set (7794 cell line-drug pairs) such that there was no overlap in the cell lines. We employed k-fold cross-validation for hyperparameter tuning and divided the training set into 5 non-overlapping folds. Our five validation sets comprised 14,761, 14,302, 14,769, 14,212 and 14,218 cell line-drug pairs, respectively. The number of features — 1429 (pathway enrichment scores and drug descriptors) were the same in training, validation and test datasets. We used Random Forest implementation from the R package ranger^52^. We performed a grid search on each fold of the training dataset. For every fold, the mtry and number of trees were varied from 1 to 10 (with the step size of 1) and 100 to 1000 (with the step size of 100), respectively. We selected the five best models with minimum Mean Squared Error (MSE) for each training data subset. Finally, we trained five models (based on pre-learned hyperparameters) on the entire training data using the parsnip R package^53^. ElasticNet was implemented using the caret^54^ and glmnet^55^ R packages. For each of the five training folds, the caret, by default, performs bootstrapping 25 times to find out the optimal model based on the minimum value of Root-Mean-Square Error (RMSE). These five optimal models were retrained on the entire training dataset.

A deep neural network (DNN) was trained using the Keras framework. The DNN architecture comprised one input layer entailing all features (pathway enrichment scores and drug descriptors) in the dataset, followed by one hidden layer of size 512, with Rectified Linear Unit (RELU) as an activation function. We kept the first two layers fixed. We used the Keras Tuner library^56^ to find the optimal set of parameters for our deep learning models. We employed Hyperband^57^ with 5-folds cross-validation on training data to find the best hyper-parameters based on validation loss. We tried the following hyper-parameters: the number of layers varied between 2 and 6, and the number of neurons, between 128 and 256 (with a step size of 4). The number of epochs was set to 30. Dropout layers were added between the layers to avoid the overfitting of the models. We tuned for drop-out rates (0.1, 0.2, 0.3, 0.4, 0.5). We used the ADAM optimizer with different learning rates (1e-3, 1e-4 and 1e-5). We optimized Mean Squared Error (MSE) as the loss function. Finally, five models were trained on the entire dataset with fold-specific tuned hyper-parameters for 50 epochs with a batch size of 128. These models were used for reporting performance on independent test data.

For the model using genes as features (instead of pathways), the only difference in the architecture was the size of the input layer, which was set to 600 (500 top highly variable genes and drug descriptors of size 100). Of note, ranger and ElasticNet were run with the same strategies discussed above.

### Benchmarking of drug response predictions

We benchmarked Precily against previously published methods – CaDRReS-Sc by Suphavilai, Chayaporn, et al.^21^ and another by Sakellaropoulos, Theodore, et al.^16^. CaDRReS-Sc is a machine learning framework that incorporates a matrix factorization-based recommender system for drug response prediction. We applied CaDRReS-Sc to the CCLE gene expression dataset and the corresponding drug response information from the GDSC database. CaDRReS-Sc was run with default settings. For the Sakellaropoulos, Theodore, et al. method, we trained models for individual drugs using the author recommended parameters, except for the variance cut-off (varcut) parameter, where we used a value of 10, to avoid late convergence. This was important because, under this strategy, we had to train drug specific models. All methods were evaluated on the same data. Final performance has been reported in terms of Pearson’s correlation for predicted vs observed IC50 value for individual drugs.

### Processing of the CTRPv2 data

CTRPv2 features a sensitivity of various well established cancer cell lines to an assorted set of small molecules comprising tool compounds, probes and drugs, including US Food and Drug Administration (FDA)-approved cancer therapeutics. We obtained SMILES for 377 compounds reported in CTRPv2^10^. For all these compounds, cell line specific IC50 values were obtained from the PharmacoGx R package^58^. We discarded IC50 outliers using the interquartile range (IQR) rule. At this stage, our data constituted 153899 drug-cell line combinations in rows and 1429 features, including pathway and drug features and LN IC50 as the response variable in columns. The remaining steps involved in evaluating the performance of Precily were kept identical as in the case of CCLE/GDSC analysis. In this analysis, 90% training dataset consisted of 138254 cell line-drug pairs and 1429 features comprising pathway enrichment scores and drug descriptors and a 10% test set (15645 cell line-drug pairs and 1429 features comprising pathway enrichment scores and drug descriptors). The five validation sets comprised 27682, 27674, 27982, 27565 and 27351 cell line-drug pairs, respectively. 1429 features were common across all five sets (pathway scores and drug descriptors), respectively.

### Training models using TCGA RNA-seq patient profiles

To predict drug response in terms of responder and non-responder, formulated as a classification problem, we utilized the H2O AutoML^40^ framework in R for training the TCGA dataset. We split this dataset in the ratio of 90% for training and 10% for testing. This 90% training dataset was subjected to the h2o.automl() function with five-fold cross-validation, ensuring there was no overlap of patients across folds and a maximum number of models was specified as 20. This resulted in automated training of 34 machine learning models (more models were automatically tested to reach convergence), including Deep learning, DRF, GBM, GLM, XGboost, XRT, and stacked ensemble models. Deep learning offered suboptimal performance (Supplementary Data 1) due to data paucity. However, we tested Precily architecture with necessary changes to make it appropriate for constructing a classifier. In this case, we used the sigmoid function in the last layer and binary cross-entropy as the loss function. The splitting of data was kept intact, as in the case of AutoML.

### Survival analysis on TCGA test dataset

The processed TCGA dataset consisted of 3108 patient-drug combinations. We conducted survival analysis on a 100% TCGA test dataset of 293 drug-patient entities. We stratified the samples using the median value of the predicted probability of the response and computed survival along with 5 year survival probability. For cox regression analysis, we used coxph() function from the survival package in R^59^.

### Performance metrics

We used two accuracy metrics to measure the performance of our models for the regression task: the coefficient of determination (R^2^) and Pearson correlation (ρ). R^2^ was calculated using the R caret^54^ package. For the TCGA dataset, we computed AUC, AUC-PR and F1 scores.

### Imputation of missing features

We used an impute^60^ package from R to impute if any pathway features are missing in the input test dataset using the nearest neighbor-based averaging approach.

### Validating CCLE cell line trained models using scRNA-seq data of cell lines

The scRNA-seq cell line pre-QC UMI count dataset comprising 30314 genes and 56982 cells encompassing 207 cell lines was obtained from Kinker, G. S. et al study^31^. This expression matrix was processed for quality control using an R script (data_processing.R) from the same study. At this stage, we were left with 53299 cells spanning 198 cell lines. This matrix was transformed into TPM by scaling with a factor of 1e6 and dividing the UMI count of genes by the total UMI count of the sample. The UMI counts are independent of gene length bias^61^. We log2-transformed this normalized matrix with the addition of a pseudo count of 1. The gene expression values of the same cell lines were averaged. This matrix was converted into pathway scores using GSVA with default parameter settings. Our final validation set consisted of 17279 drug cell line combinations entailing 116 cell lines that overlapped with the cell lines featured in the GDSC2 dataset and tested against 173 drugs.

### Predicting the response of paclitaxel drug in MDA-MB-231 single cells using CCLE cell line trained models

Lee et al. dataset was obtained by processing FASTQ files of MDA-MB-231 cells from SRA id SRP040309. We align FASTQ files using STAR aligner^48^ with GRCh37 reference genome and GRCh37 GTF file from Ensembl (release 75)^62^. To estimate gene expression, we used HTSeq-count^63^. The HTSeq generated read counts were transformed into TPM values by dividing raw counts by gene length, then scaling with a factor of 1e6 and dividing by total read counts in the sample. We discarded the genes for which gene length was not retrieved by the EDASeq R package^64^. This was followed by the conversion of Ensembl gene IDs to official gene symbols using Homo_sapiens.GRCh37.75.gtf annotation file. We retained genes having a TPM of at least 1 in at least 10% of samples. This matrix was subjected to log2 transformation with the addition of pseudo count 1. We averaged gene expression values of five samples each of naive, stressed, and cells more sensitive to paclitaxel, respectively and converted them into pathway scores using GSVA. We predicted drug response for treatment-naive and population of cells more sensitive to paclitaxel in terms of LN IC50 values.

### Validating TCGA data trained models using BRAF-mutant melanoma patient profiles

We used publicly available Reads Per Kilobase of transcript per million mapped reads (RPKM) RNA-seq profiles of BRAF mutant melanoma patients for drug response prediction. This dataset was comprised of six paired patients for which RNA-seq was performed before and after treatment with RAF or RAF + MEK inhibitors during disease progression. This dataset (Wagle, Nikhil, et al. dataset^41^) also included therapy response information. For our analysis, we transformed RPKM normalized RNA-seq profiles to TPM by dividing each RPKM value by the sum of the RPKM values for all genes in the sample and multiplying by one million^65^. The TPM normalized matrix was log2 transformed with the addition of a pseudo count of 1. The resulting matrix was transformed into pathway scores using GSVA. For the three patients for whom information on the mechanism of acquired resistance to dabrafenib/trametinib was available, we predicted a therapeutic response to dabrafenib and trametinib.

### Data visualization

For data visualization, plots were generated in Prism 9.0.0 (GraphPad) and in R with ggplot2 (3.3.5), pheatmap (1.0.12), ggpubr (0.4.0) and ggridges (0.5.3).

### Reporting summary

Further information on research design is available in the Nature Research Reporting Summary linked to this article.

## Supplementary information

Supplementary information

Description of Additional Supplementary Files

Dataset 1

Dataset 2

Reporting Summary

## Data Availability

The bulk RNA-seq data of 5 PCa cell lines (LNCaP, VCaP, DU145, PC3, DUCAP) generated in this study have been deposited in the NCBI GEO database under accession code GSE211721. The bulk RNA-seq data of the LNCaP cell line after treatment with AR antagonists generated in this study have been deposited in the NCBI GEO database under accession code GSE211781. The bulk RNA-seq data of LNCaP xenografts comprising 54 samples spanning different treatment groups (PRE-CX, POST-CX, CRPC, ENZS and ENZR) generated in this study have been deposited in the NCBI GEO database GSE211856. The CCLE, GDSC, TCGA, and CTRPv2 datasets are publicly available. The CCLE RNA-seq dataset was downloaded from https://sites.broadinstitute.org/ccle/. Drug response data was sourced from the GDSC website https://www.cancerrxgene.org/. TCGA data were downloaded from Broad GDAC Firehose (https://gdac.broadinstitute.org/). The drug response information for the CTRPv2 dataset (https://portals.broadinstitute.org/ctrp.v2.1/) was obtained from the R package PharmacoGx. The pre-QC UMI count scRNA-seq cell line data was obtained from Broad Institute’s single cell portal accession number SCP542 (requires login). Another scRNA-seq data of MDA-MB-231 cell line under differential treatment conditions was obtained from accession no. SRP040309. Bulk RNA-seq data of melanoma was obtained from NCBI GEO GSE77940. To compute pathway enrichment scores, we downloaded a collection of canonical pathways (v.6.1) from MsigDB (http://www.gsea-msigdb.org/gsea/msigdb). Source data are provided with this paper.

## Source data

Source Data

## Code availability

All source codes are available at Github https://github.com/SmritiChawla/Precily which is linked with Zenodo 10.5281/zenodo.7024834^66^.
