# Supplementary material for: Gene expression based inference of cancer drug sensitivity
Source: Nat Commun. 2022 Sep 27;13:5680. doi: 10.1038/s41467-022-33291-z (PMC9515171; doi:10.1038/s41467-022-33291-z)
Supplement: Supplementary file 1 — Supplementary information [file 41467_2022_33291_MOESM1_ESM.pdf]

Supplementary Information

Gene expression based inference of cancer drug sensitivity

Smriti Chawla[1], Anja Rockstroh[2], Melanie Lehman[2,3], Ellca Ratther[2], Atishay Jain[4], Anuneet Anand[4], Apoorva Gupta[5], Namrata Bhattacharya[2,4], Sarita Poonia[1], Priyadarshini Rai[1], Nirjhar Das[6], Angshul Majumdar[4,7,8], Jayadeva[6], Gaurav Ahuja[1], Brett G. Hollier[2], Colleen C. Nelson[2*], Debarka Sengupta[1,4,7*]

1. Department of Computational Biology, Indraprastha Institute of Information Technology-Delhi (IIIT-Delhi), Okhla, Phase III, New Delhi-110020, India.
2. Australian Prostate Cancer Research Centre-Queensland, Faculty of Health, School of Biomedical Sciences, Centre for Genomics and Personalised Health, Queensland University of Technology, Translational Research Institute, Brisbane, Australia.
3. Vancouver Prostate Centre, Department of Urologic Sciences, University of British Columbia, Vancouver, Canada.
4. Department of Computer Science and Engineering, Indraprastha Institute of Information Technology-Delhi (IIIT-Delhi), Okhla, Phase III, New Delhi-110020, India.
5. Department of Biotechnology, Delhi Technological University, Shahbad Daulatpur, Main Bawana Road, Delhi-110042, India.
6. Department of Electrical Engineering, Indian Institute of Technology Delhi, Hauz Khas, Delhi, 110016, India.
7. Centre for Artificial Intelligence, Indraprastha Institute of Information Technology-Delhi (IIIT-Delhi), Okhla, Phase III, New Delhi-110020, India.
8. Department of Electronics & Communications Engineering, Indraprastha Institute of Information Technology-Delhi (IIIT-Delhi), Okhla, Phase III, New Delhi-110020, India.

**Corresponding authors: {debarka@iiitd.ac.in, colleen.nelson@qut.edu.au}.*

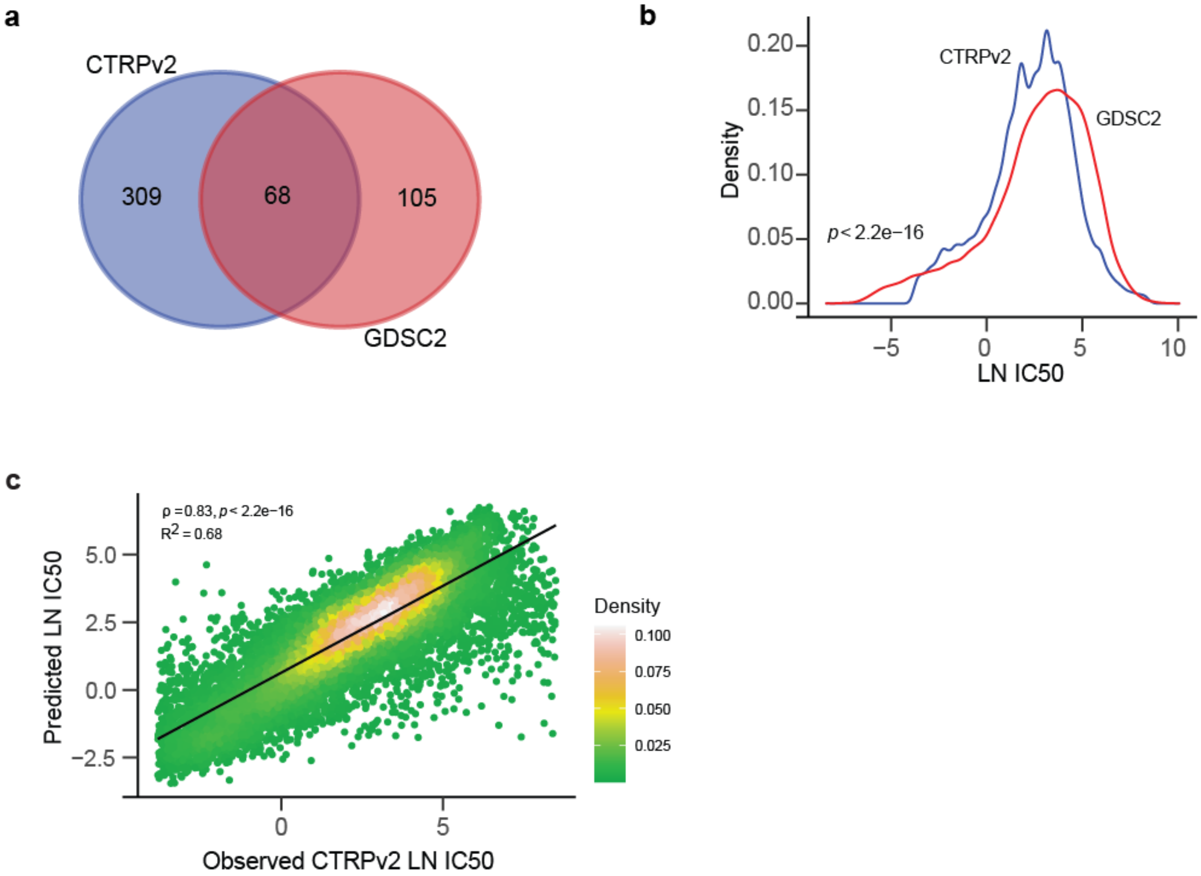

Supplementary Fig. 1. a Venn diagram depicting common drugs between CTRPv2 and GDSC2 datasets. **b** The density plot showing a comparison of the distribution of LN IC50 values from CTRPv2 and GDSC2 datasets. Wilcoxon rank-sum test (two-sided) indicates the substantial difference between the two distributions, suggesting that combining both databases could be misleading. **c** Scatterplot of observed and predicted LN IC50 showing prediction performance of CaDRReS-Sc on models trained on combined CCLE and CTRPv2 datasets. Notably, after Precily, CaDRReS-Sc reported the best performance on the CCLE/GDSC data. P-value was calculated using a two-sided t-test. Source data are provided in the Source Data file.

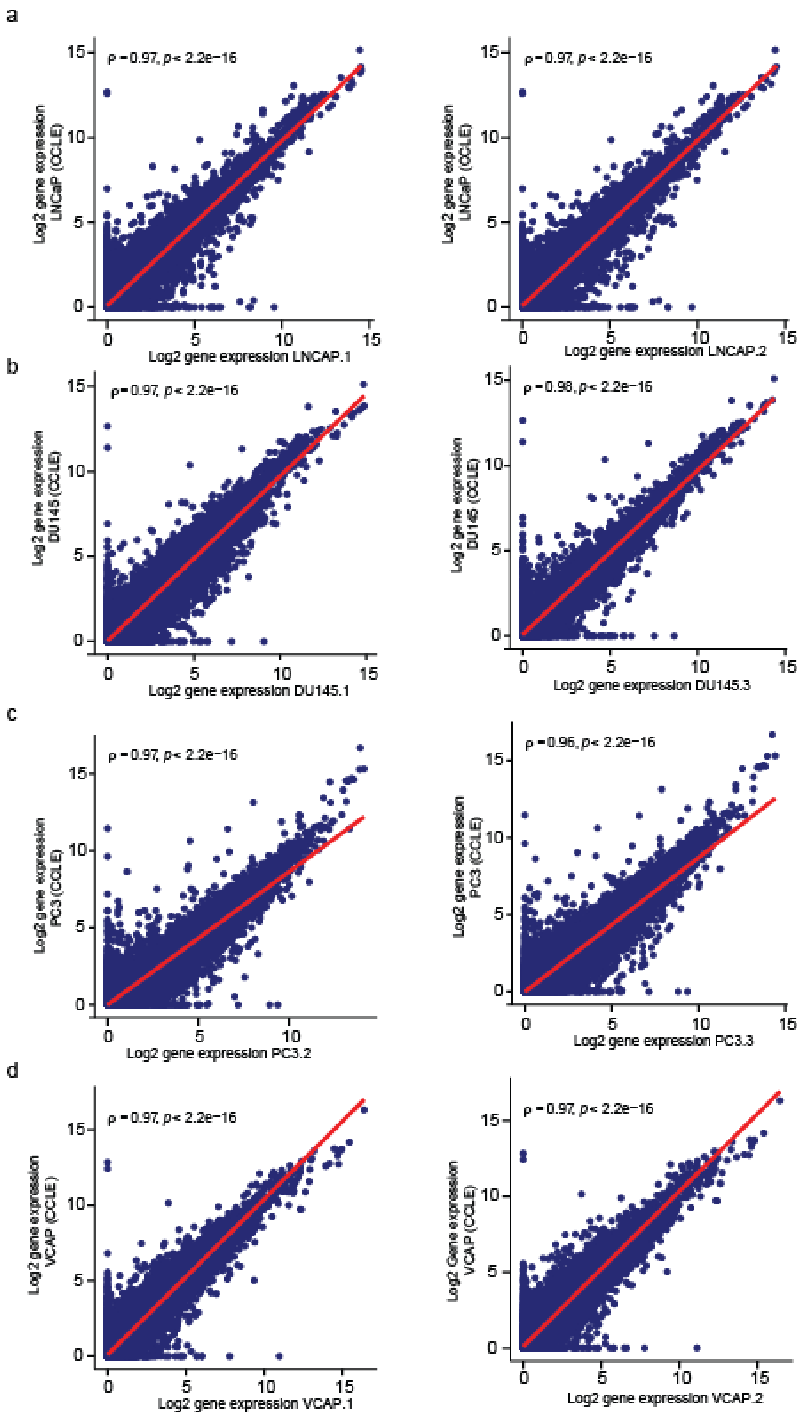

Supplementary Fig. 2a-d. Scatter plots demonstrating the correlation between gene expression profiles for two biological replicates of our in-house PCa cell lines (LNCaP, DU145, PC3 and VCAP) and CCLE gene expression profiles. P-value was calculated using a two-sided t-test. The red lines in the scatter plots represent the respective regression lines. Source data are provided in the Source Data file.

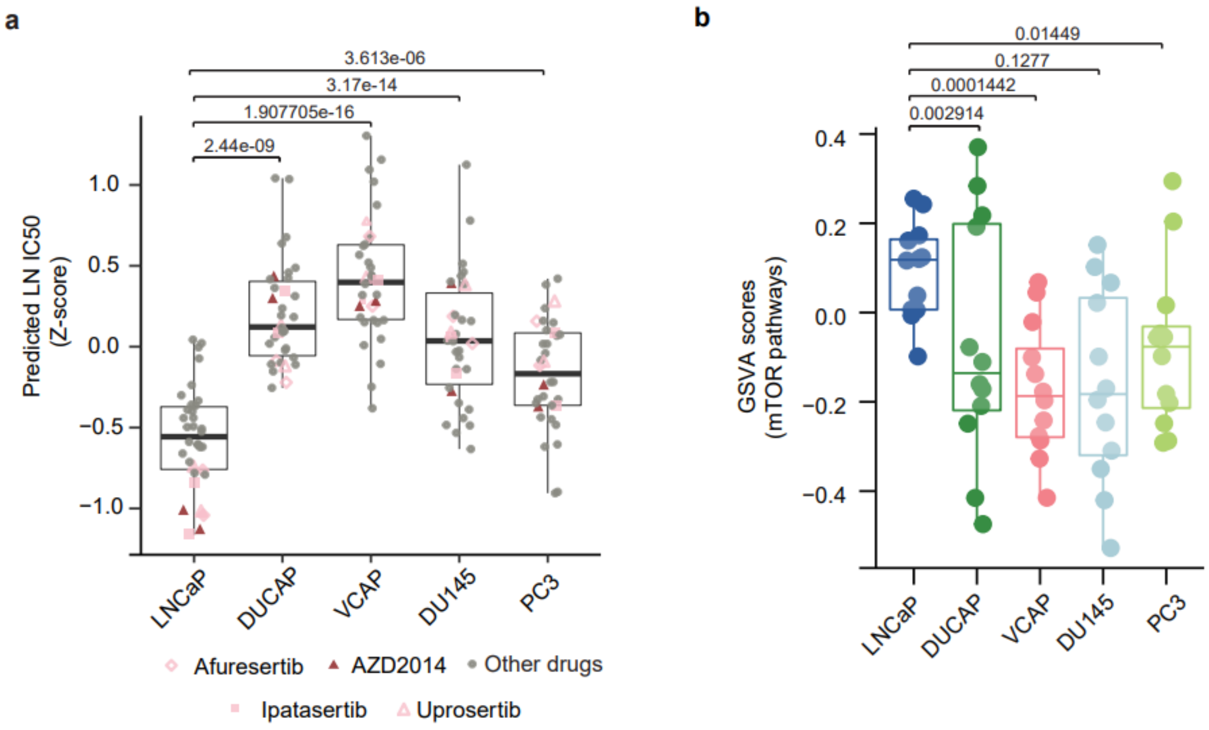

Supplementary Fig. 3. LNCaP cell line sensitivity to drugs targeting mTOR signaling. a Boxplots depicting the distribution of drug response prediction of mTOR/PI3K signaling targeting drugs. LNCaP cell line was predicted to be more sensitive to these drugs, with ipatasertib and AZD2014 having the most profound effect. Afuresertib, ipatasertib and uprosertib are depicted using pink colored diamond, filled square and empty triangle, respectively. AZD2014 is denoted using darkred small filled triangle, and other drugs are represented using a grey filled circles. Each dot in the boxplot represents the predicted LN IC50 (Z-score) relating to n=5 PCa cell lines. We examined n=17 drugs and n=2 biological replicates of each cell line. P-values were calculated using a two-sided Wilcoxon rank-sum test. **b** Boxplots showing the distribution of GSVA scores of mTOR-related pathways highlighting increased expression of these pathways in the LNCaP cell line. Each dot in the boxplot represents a pathway enrichment score relating to n=5 PCa cell lines. We examined n=6 pathways and n=2 biological replicates of each cell line. P-values were calculated using a two-sided Wilcoxon rank-sum test. In all boxplots, the middle horizontal line represents the median value. Each box spans the lower quartile to the upper quartile. The whiskers indicate the minimum and maximum values within 1.5 times the IQR. Source data are provided in the Source Data file.

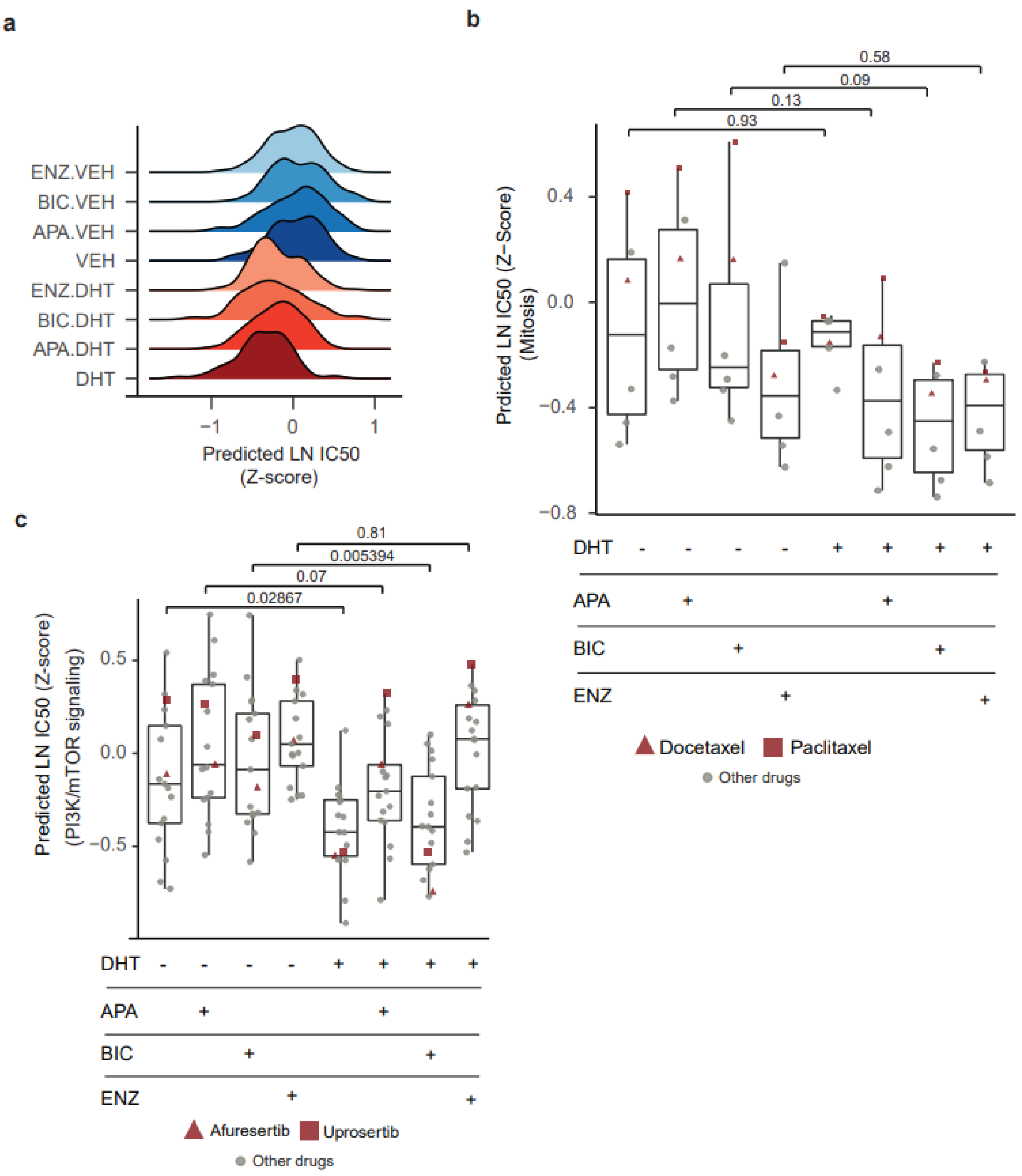

Supplementary Fig. 4. Drug response prediction and analysis in LNCAP cells under different treatment conditions. **a** Ridgeplot showing the overall distribution of predicted LN IC50 (Z-score) of 155 drugs tested against PCa cell lines in the GDSC2 dataset across the treatment conditions. **b** Boxplot showing predicted LN IC50 (Z-score) of drugs targeting mitosis, specifically highlighting docetaxel and paclitaxel. Docetaxel and paclitaxel are depicted using darkred colored filled triangle and square respectively. Other drugs are represented using a grey filled circles. We examined n=6 drugs across n=8 treatment conditions (DHT, BIC.DHT, ENZ.DHT, APA.DHT, VEH, BIC.VEH, ENZ.VEH and APA.VEH). P-values were calculated using a two-sided Wilcoxon rank-sum test. **c** Boxplot depicting predicted LN IC50 (Z-score) of drugs targeting PI3K/mTOR pathway highlighting afuresertib and uprosertib. Afuresertib and uprosertib are depicted using darkred colored filled triangle and square respectively. Other drugs are represented using a grey filled circles. P-values were calculated using a two-sided Wilcoxon rank-sum test. We examined n=17 drugs across all treatment conditions (n=8). In all boxplots, the middle horizontal line represents the median value. Each box spans the lower quartile to the upper quartile. The whiskers indicate the minimum and maximum values within 1.5 times the IQR. Source data are provided in the Source Data file.

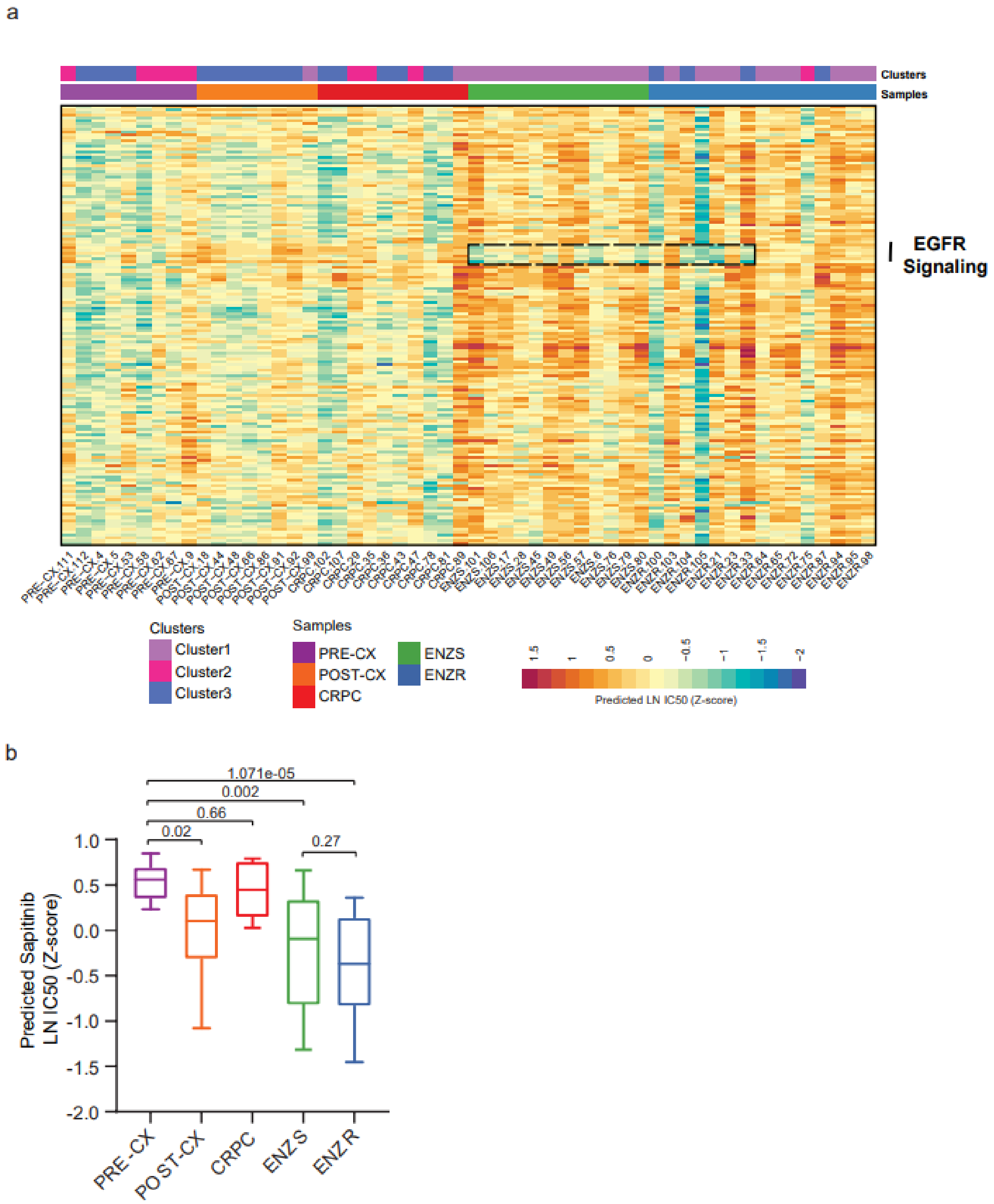

Supplementary Fig. 5. Predictions in LNCAP xenografts under different treatment conditions. a Visualization of predicted LN IC50 (Z-score) in 54 xenograft tumor samples for 155 GDSC drugs. Heatmap showing the sensitivity of some ENZ treated tumors to 'EGFR signaling' targeting drugs. **b** Boxplot of predicted LN IC50 (Z-score) of sapitinib across the tumor types revealing the highest sensitivity of this drug for ENZR tumors. We examined n=9 PRE-CX, n=8 POST-CX, n=10 CRPC, n=12 ENZS and n=15 ENZR samples. P-values were calculated using a two-sided Wilcoxon rank-sum test. Each box spans the lower quartile to the upper quartile. In all boxplots, the middle horizontal line represents the median value. The whiskers indicate the minimum and maximum values within 1.5 times the IQR. Source data are provided in the Source Data file.

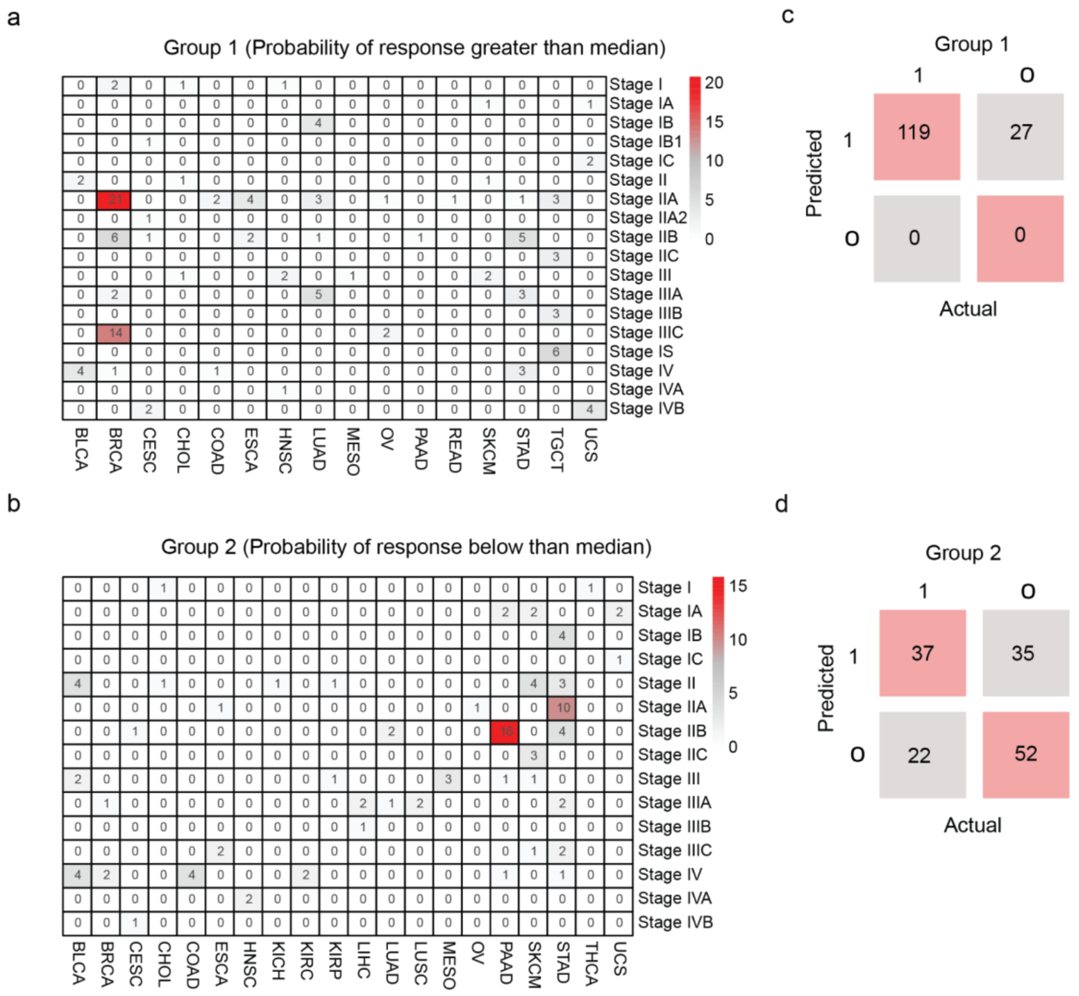

Supplementary Fig. 6. a & b Heatmaps showing the frequency distribution of stages across multiple cancer types for group 1 and group 2, respectively, stratified based on the median value of the probability of response as predicted through a classifier trained on the TCGA dataset. We have removed those samples where stage information is not available. **c** Confusion matrix showing classification results for group 1. Our median value of 0.63 resulted in group 1 containing responders (class 1 as per classification problem) alone. **d** Confusion matrix showing classification results for group 2. The actual class depicts treatment response information from TCGA clinical metadata files. The treatment response information is categorized as responder (label=1) for complete response and the partial response of patients. Clinical progressive disease and stable disease in patients represent non-responder categories (label=0). Source data are provided in the Source Data file.

Cancer	#Samples	#Drugs
ALL	13	156
BLCA	16	156
BRCA	44	171
CESC	2	155
CLL	2	155
COREAD	41	160
DLBC	11	156
ESCA	22	156
GBM	18	156
HNSC	11	156
KIRC	12	156
LAML	14	155
LCML	9	156
LGG	6	155
LIHC	14	156
LUAD	48	156
LUSC	14	156
MB	3	156
MESO	6	155
MM	14	156
NB	9	156
OV	18	156
PAAD	26	156
PRAD	5	155
SCLC	30	156
SKCM	22	156
STAD	19	156
THCA	8	156
UCEC	8	156
UNCLASSIFIED	85	170

Supplementary Table 1. Overall summary of the CCLE and GDSC dataset. The table shows the frequency of cell lines (n=550) from the CCLE database and the frequency of tested drugs (n=173) from the GDSC database spanning 29 classified cancer types used for the training dataset.

Cancer	#Samples	#Drugs
ACC	3	9
BLCA	91	18
BRCA	209	31
CESC	78	14
CHOL	8	4
COAD	44	11
ESCA	40	14
HNSC	80	13
KICH	2	2
KIRC	12	11
KIRP	10	11
LGG	140	24
LIHC	22	11
LUAD	98	17
LUSC	63	15
Meso	35	18
OV	7	6
PAAD	75	16
PCPG	2	6
PRAD	38	10
READ	14	8
SARC	59	28
SKCM	52	34
STAD	132	26
TGCT	59	7
THCA	12	3
THYM	3	5
UCEC	19	6
UCS	36	9

Supplementary Table 2. Description of the TCGA dataset. The table shows the frequency of patients (n=1443) from the GDAC firehose database and the frequency of tested drugs (n=139) from TCGA clinical response data spanning 29 classified cancer types used for the training dataset.

Supplementary note 1: Clinical characteristics of melanoma patients

Patient 1. The patient was diagnosed with Stage IIIC melanoma. After one year of initial treatment, the patient again showed signs of recurrent disease and underwent pre-treatment biopsy and intensity-modulated radiation therapy (IMRT). This patient exhibited both BRAF V600E and V600K mutations and was recruited into a Dabrafenib and Trametinib phase I/II study. After three months, the patient was removed from the study due to the development of resistance. Then the patient was treated with an anti-PDL1 antibody for four months until progression and subsequently treated with four cycles of Ipilimumab. The patient died of his condition approximately nine months after the discontinuation of RAF and MEK inhibitors[1].

Patient 2. Patient 2, a 48-year old man initially diagnosed with stage IB melanoma developed extensively metastatic melanoma after five years of initial diagnosis, confirmed by a pleural biopsy (pre-treatment). Further, clinical mutation analysis revealed the presence of BRAF V600E mutation. The patient was subjected to first-line treatment of Dabrafenib and Trametinib which showed partial response but after three months, routine scans revealed significant disease progression. The potential cause of resistance to therapy was the presence of BRAF splice variant as detected by RNA-seq and whole exome sequencing (WES) in post-treatment tumors but not in pre-treatment tumors. The patient died six months after being diagnosed with metastatic disease[1].

Patient 3. Patient 3, a 42-year old man underwent surgery and lymph node dissection of stage IIIC melanoma of the left thigh. The patient had a BRAF V600E mutation as revealed by clinical mutational analysis. After six months of surgery, the patient was subjected to first-line therapy of dabrafenib and trametinib. But after nearly one year the patient developed progressive disease and the potential underlying cause of acquired resistance to therapy was the presence of BRAF amplification in post-treatment tumors as determined by WES. The patient was given Ipilimumab for a short time but died after four cycles and three months after discontinuation of dabrafenib and trametinib therapy[1].

References

1. Wagle, N. *et al.* MAP Kinase Pathway Alterations in *BRAF*-Mutant Melanoma Patients with Acquired Resistance to Combined RAF/MEK Inhibition. *Cancer Discovery* vol. **4** 61–68 (2014).
