# Supplementary material for: Gene expression based inference of cancer drug sensitivity
Source: Nat Commun. 2022 Sep 27;13:5680. doi: 10.1038/s41467-022-33291-z (PMC9515171; doi:10.1038/s41467-022-33291-z)
Supplement: Supplementary file 2 — Description of Additional Supplementary Files [file 41467_2022_33291_MOESM2_ESM.pdf]

Description of Additional Supplementary Files

File name: **Supplementary Data 1**
Description: Performance evaluation of AutoML models. Performance was determined using AUC-PR, AUC and F1 scores.

File name: **Supplementary Data 2**
Description: Result of predictions for BRCA tumor-drug pairs. This file also includes information on patients' overall survival, groups used for survival analysis and observed clinical responses.
