# Supplementary material for: Gene expression based inference of cancer drug sensitivity
Source: Nat Commun. 2022 Sep 27;13:5680. doi: 10.1038/s41467-022-33291-z (PMC9515171; doi:10.1038/s41467-022-33291-z)
Supplement: Supplementary file 5 — Reporting Summary [file 41467_2022_33291_MOESM5_ESM.pdf]

nature portfolio

Corresponding author(s): Debarka Sengupta

Last updated by author(s): 2022/09/8

Reporting Summary

Nature Portfolio wishes to improve the reproducibility of the work that we publish. This form provides structure for consistency and transparency in reporting. For further information on Nature Portfolio policies, see our Editorial Policies and the Editorial Policy Checklist.

Statistics

For all statistical analyses, confirm that the following items are present in the figure legend, table legend, main text, or Methods section.

n/a	Confirmed	
☐	☒	The exact sample size (*n*) for each experimental group/condition, given as a discrete number and unit of measurement
☐	☒	A statement on whether measurements were taken from distinct samples or whether the same sample was measured repeatedly
☐	☒	The statistical test(s) used AND whether they are one- or two-sided<br>*Only common tests should be described solely by name; describe more complex techniques in the Methods section.*
☐	☒	A description of all covariates tested
☒	☐	A description of any assumptions or corrections, such as tests of normality and adjustment for multiple comparisons
☐	☒	A full description of the statistical parameters including central tendency (e.g. means) or other basic estimates (e.g. regression coefficient) AND variation (e.g. standard deviation) or associated estimates of uncertainty (e.g. confidence intervals)
☐	☒	For null hypothesis testing, the test statistic (e.g. *F*, *t*, *r*) with confidence intervals, effect sizes, degrees of freedom and *P* value noted<br>*Give P values as exact values whenever suitable.*
☒	☐	For Bayesian analysis, information on the choice of priors and Markov chain Monte Carlo settings
☒	☐	For hierarchical and complex designs, identification of the appropriate level for tests and full reporting of outcomes
☐	☒	Estimates of effect sizes (e.g. Cohen's *d*, Pearson's *r*), indicating how they were calculated

Our web collection on statistics for biologists contains articles on many of the points above.

Software and code

Policy information about availability of computer code

Data collection	Applied Biosystems® 3500 Genetic Analyzer
Data analysis	R-4.1.2, Python 3.9.7, GraphPad Prism 9, keras 2.8.0, keras-tuner 1.1.0, caret(v6.0.90), glmnet(v4.1.3), ranger(v0.13.1), GSVA(v1.40.1), GeneMapper® v5.0 software, h2o(v3.36.0.4), PubChemPy(v1.0.4), SMILESVec (https://github.com/hkmztrk/SMILESVecProteinRepresentation), EDASeq(v2.26.1), impute(v1.66.0), STAR-2.7.9a, Survival (v3.2.13), HTSeq-0.12.4, ggpubr(v0.4.0), FastQC(v.0.11.9), PharmacoGx(v2.6.0), ggplot2(v3.3.5), pheatmap(v1.0.12), parsnip(v0.2.1), ggridges(v0.5.3), TrimGalore (v0.11.2), STAR-2.4.0j, RSEM(v1.2.18). Codes for reproducibility are deposited in: https://github.com/SmritiChawla/Precily.

For manuscripts utilizing custom algorithms or software that are central to the research but not yet described in published literature, software must be made available to editors and reviewers. We strongly encourage code deposition in a community repository (e.g. GitHub). See the Nature Portfolio guidelines for submitting code & software for further information.

Data

Policy information about availability of data

All manuscripts must include a data availability statement. This statement should provide the following information, where applicable:

- Accession codes, unique identifiers, or web links for publicly available datasets
- A description of any restrictions on data availability
- For clinical datasets or third party data, please ensure that the statement adheres to our policy

The bulk RNA-seq data of 5 PCa cell lines (LNCaP, VCaP, DU145, PC3, DUCAP) generated in this study have been deposited in the NCBI GEO database under accession code GSE211721 [https://www.ncbi.nlm.nih.gov/geo/query/acc.cgi?acc=GSE211721]. The bulk RNA-seq data of the LNCaP cell line after treatment with AR antagonists generated in this study have been deposited in the NCBI GEO database under accession code GSE211781 [https://www.ncbi.nlm.nih.gov/geo/query/

acc.cgi?acc=GSE211781]. The bulk RNA-seq data of LNCaP xenografts comprising 54 samples spanning different treatment groups (PRE-CX, POST-CX, CRPC, ENZS and ENZR) generated in this study have been deposited in the NCBI GEO database GSE211856 [https://www.ncbi.nlm.nih.gov/geo/query/acc.cgi?acc=GSE211856]. The CCLE, GDSC, TCGA, and CTRPv2 datasets are publicly available. The CCLE RNA-seq dataset was downloaded from https://sites.broadinstitute.org/ccle/. Drug response data was sourced from the GDSC website https://www.cancerrxgene.org/. TCGA data were downloaded from Broad GDAC Firehose (https://gdac.broadinstitute.org/). The drug response information for the CTRPv2 dataset (https://portals.broadinstitute.org/ctrp.v2.1/) was obtained from the R package PharmacoGx. The pre-QC UMI count scRNA-seq cell line data was obtained from Broad Institute's single cell portal accession number SCP542 (requires login). Another scRNA-seq data of MDA-MB-231 cell line under differential treatment conditions was obtained from accession no. SRP040309 [https://www.ncbi.nlm.nih.gov/sra/SRP040309]. Bulk RNA-seq data of melanoma was obtained from NCBI GEO GSE77940 [https://www.ncbi.nlm.nih.gov/geo/query/acc.cgi?acc=GSE77940]. To compute pathway enrichment scores, we downloaded a collection of canonical pathways (v.6.1) from MsigDB (http://www.gsea-msigdb.org/gsea/msigdb). Source data are provided with this paper.

Field-specific reporting

Please select the one below that is the best fit for your research. If you are not sure, read the appropriate sections before making your selection.

☒ Life sciences ☐ Behavioural & social sciences ☐ Ecological, evolutionary & environmental sciences

For a reference copy of the document with all sections, see nature.com/documents/nr-reporting-summary-flat.pdf

Life sciences study design

All studies must disclose on these points even when the disclosure is negative.

Sample size	Sample sizes were determined based on data availability, following similar studies from the past. No additional sample size estimation was performed. The reasonably consistent performance of our method on several datasets further demonstrated that sample sizes were sufficient.
Data exclusions	No data excluded.
Replication	Two biological replicates of each of the untreated prostate cancer cell lines were analyzed. We observed similar trend in results in two biological replicates for untreated prostate cancer cell lines. For LNCaP cell line under different treatment conditions, averaged GSVA scores for the two biological replicates for each condition were used for downstream analysis. For the prostate cancer xenograft experiment, we had 8-15 biological repeats per sample group. Prostate cancer xenograft study was one large study and the biological replication is represented by having multiple animals within the same tumor group/phenotype (n of 9 -15 individual mice/tumors per group). The RNA-seq data presented in the manuscript is from 54 individual tumors collected as part of a larger cohort of ~90 tumors/animals undergoing the identical sequential treatment arms. Due to the cost of sequencing and/or poor RNA quality, RNA-seq was not performed on all tumors from the larger cohort. However, the biological phenotypes of tumors (e.g. take rate, growth kinetics, response to Castration and ENZ treatment) are highly consistent with other similar studies performed within the laboratories of Prof Nelson and Dr Brett Hollier over the last 5 years and those published by external colleagues previously (Bishop, JL et al., Cancer Discovery 2017 Jan;7(1):54-71. doi: 10.1158/2159-8290.CD-15-1263. Epub 2016 Oct 26; Toren, P et al., Eur Urol 2015 Jun;67(6):986-990. doi: 10.1016/j.eururo.2014.08.006. Epub 2014 Aug 20; Locke, JA et al., Cancer Res 2008 Aug 1;68(15):6407-15. doi: 10.1158/0008-5472.CAN-07-5997).
Randomization	For every machine learning task, we used 5 fold cross validation. As the strictest possible measure, we performed cell line/patient wise split ensuring none of train, test and validation have overlapping samples (cell line, tumor). For the xenograft studies, mice were randomized into the treatment groups.
Blinding	Performance of machine learning tasks has been reported on blinded test dataset. For other experiments blinding was not relevant because gene expression was quantified for all the samples/cell lines/tumor.

Reporting for specific materials, systems and methods

We require information from authors about some types of materials, experimental systems and methods used in many studies. Here, indicate whether each material, system or method listed is relevant to your study. If you are not sure if a list item applies to your research, read the appropriate section before selecting a response.

Materials & experimental systems

n/a	Involved in the study	
☒	☐	Antibodies
☐	☒	Eukaryotic cell lines
☒	☐	Palaeontology and archaeology
☐	☒	Animals and other organisms
☒	☐	Human research participants
☒	☐	Clinical data
☒	☐	Dual use research of concern

Methods

n/a	Involved in the study	
☒	☐	ChIP-seq
☒	☐	Flow cytometry
☒	☐	MRI-based neuroimaging

Eukaryotic cell lines

Policy information about cell lines

Cell line source(s)	LNCaP (#CRL-1740™ clone FGC), VCaP (#CRL-2876), PC3 (#CRL-1435), DU145 (#HTB-81) were purchased from the American Type Culture Collection (ATCC), DUCAP line (fibroblast free) was a generous gift from M. Ness (VTT Technical Research Centre of Finland)
Authentication	In house Cell Line Authentication Service at the Queensland University of Technology (QUT) Genomics Research Centre, Nine short tandem repeat (STR) loci plus the gender determining locus Amelogenin, were amplified using the commercially available GenePrint® 10 System kit from Promega. The cell line samples were processed using the Applied Biosystems® 3500 Genetic Analyzer. Data were analysed using GeneMapper® v5.0 software (Applied Biosystems). Cell lines were authenticated using STR analysis as described in 2012 in ANSI Standard (ASN-0002) by the ATCC Standards Development Organization. STR profiles of query samples are compared to the ATCC STR Database to verify cell line identity. Cell lines with ≥80% match are considered to be related. All cell lines used in the manuscript had ≥80% matches to their database entries.
Mycoplasma contamination	All cell lines tested negative for mycoplasma
Commonly misidentified lines (See ICLAC register)	No commonly misidentified cell lines used in this study.

Animals and other organisms

Policy information about studies involving animals; ARRIVE guidelines recommended for reporting animal research

Laboratory animals	Five-six week old adult male NOD-SCID mice were used in the prostate cancer xenograft study.
Wild animals	The study did not involve wild animals.
Field-collected samples	The study did not involve samples collected from the field.
Ethics oversight	Animal studies were performed with the approval of the University of Queensland and Queensland University of Technology (QUT) Animal Ethics Committees (ethics approval number QUT/572/17) and in accordance with accepted standards of humane animal care as outlined in the 'Australian Code of Practice for the Care and Use of Animals for Scientific Purposes' and the universities' guidelines for the use of laboratory animals.

Note that full information on the approval of the study protocol must also be provided in the manuscript.
